# Multilayer Spintronic THz Emitters for On-Chip Ultrafast Current Generation

**DOI:** 10.1021/acs.nanolett.6c02697

**Published:** 2026-10-22

**Authors:** Wolfgang Hoppe, Mohamed Amine Wahada, Paul Marschall, James Caleb Peters, Stuart S. P. Parkin, Georg Woltersdorf

**Affiliations:** † Institute of Physics, Martin Luther University Halle-Wittenberg, von Danckelmann Platz 3, 06120 Halle, Germany; ‡ Max Planck Institute for Microstructure Physics, Weinberg 2, 06120 Halle, Germany; ¶ Cluster of Excellence - Center for Chiral Electronics, Halle-Berlin-Regensburg, Germany

**Keywords:** spin Hall effect, ultrafast demagnetization, THz currents, nanostructures, electro-optic sampling, THz emitter

## Abstract

Spintronic terahertz (THz) emitters based on ultrafast spin-to-charge conversion in heavy metal | ferromagnet heterostructures are compact, broadband THz sources with bandwidths exceeding conventional semiconductor-based emitters. However, achievable current amplitudes per unit area are fundamentally limited by spin-current transport across a single heavy metal | ferromagnet interface. Here, we demonstrate a multilayer spintronic THz emitter architecture comprising repeated heavy metal | ferromagnet bilayers separated by ultrathin MgO barriers, enabling constructive addition of generated charge currents. Using GHz-bandwidth electrical sampling and THz-bandwidth electro-optic sampling, we observe monotonic amplitude enhancement with increasing layer number. This scaling behavior is quantitatively captured by a model incorporating optical absorption and impedance matching effects. Finally, by integrating multilayer emitters into coplanar waveguide structures, we demonstrate on-chip THz current generation and propagation. These results establish multilayer spintronic THz emitters as a scalable platform for ultrafast current generation and integrated on-chip THz electronics.

## Graphical abstract

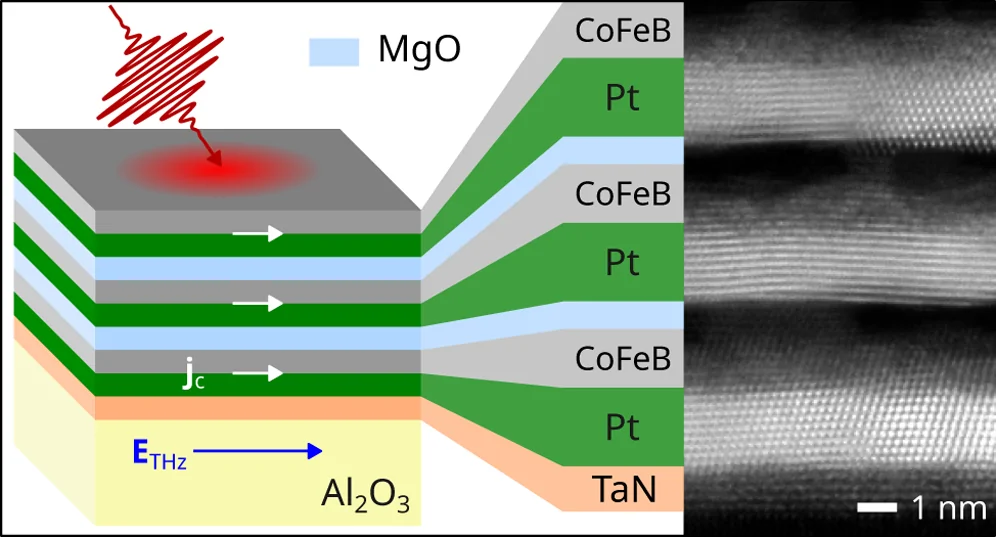

Electromagnetic waves at THz frequencies, ranging from 0.1 THz to 30 THz, have proven to be extremely important for numerous applications such as broadband wireless communication, material analysis, and security technology.^2–4^ Unfortunately, the generation of THz radiation can be rather challenging. Available sources typically involve nonlinear optical difference frequency generation, semiconductor-based photoswitches, or plasma sources - all of which are driven by pulsed laser sources operating in the fs regime.^5–10^ Due to numerous low energy excitation bands in solid materials (phonons, magnons, etc.) THz radiation tends to be strongly absorbed in the generating crystals in most cases greatly limiting the THz generation bandwidth. Recently, it was discovered that layer stacks of ultrathin magnetic and nonmagnetic metals (few nm thick) can act as efficient broadband THz emitters (0.1 THz to 30 THz).^11^ In these metallic multilayer structures an optical pulse results in ultrafast demagnetization of the ferromagnetic layer (FM).^12–18^ In this process a fraction of the excess angular momentum propagates in the form of a spin current into the adjacent heavy metal layer (HM) where it is converted to an in-plane charge current via the inverse spin Hall effect (ISHE) leading to the radiation of electromagnetic THz pulses.^11,19–24^ Here the bandwidth is not limited by the generating materials due to the small thicknesses of the layer stacks. Such emitters have by now been optimized for various purposes and are widely used for ultrafast spectroscopy.^25–34^

Typical metallic materials for the FM are e.g. Fe, Ni_80_Fe_20_, or Co_60_Fe_20_B_20_ (CoFeB) and for the HM e.g. W, Ta, or Pt.^21^ In a previous work^35^ it was shown that in principle the Spintronic Terahertz Emitter (STE) can be incorporated as on-chip source for ultrafast current pulses and was probed with a bandwidth of 50 GHz using an oscilloscope. Furthermore, it should be noted that STEs are easily fabricated on any substrate, making them promising for applications in THz spectroscopy, imaging, and communications.^35^ So far, the use of STEs has been largely limited to ultra broadband spectroscopic applications, e.g. to probe spin current propagation across tunnel barriers and in metallic spacer layers.^36–38^ A limitation for the maximum amplitude that can be generated by STEs per unit area is the fact that the spin current that has to be transported across the HM | FM interface is limited by the interface conductance and the demagnetization rate. A way to address this limitation is the parallel excitation of several stacked STEs separated each by a dielectric spacer. Approaches so far include, either a thick spacer (∼110 nm SiO_2_) acting as a photonic crystal and thus enhancing the absorbed pump light^39^ or a thin spacer (∼2 nm MgO), where all individual STE radiate in phase.^40^ Both methods lead to an increased electric far-field amplitude of about 1.7.

In this study we follow a similar approach as Yang et al.^40^ by using a dielectric barrier of only 0.7 nm MgO which has been proven to be sufficient to prevent spin current propagation.^36^ In contrast, to earlier works,^39,40^ we quantify and optimize the THz near-field amplitude of such emitters from DC to 1 THz. This is achieved by utilizing the complementary methods of fast GHz electronic sampling as well as THz-bandwidth electro-optic sampling. Additionally, we incorporate our multilayer STEs into Au waveguide structures and investigate the on-chip propagation.

In the following intense light pulses from an amplified femtosecond laser system (1030 nm, FWHM pulse width 300 fs, repetition rate 50 kHz, maximum used average power 620 mW) are used to optically pump the STE, as these pulses demagnetize the FM layer (CoFeB). Consequently, spin current pulses **j**_s_(τ) are launched into the HM layer (Pt) where they are converted into in-plane charge current pulses **j**_c_(τ) via an ISHE. An electrical coplanar probe tip with a 50 GHz bandwidth is used to pick-up and propagate the low frequency tail of the resulting THz-current pulse *I*(τ), which is then detected using a synchronized sampling oscilloscope.^35^ This current pulse corresponds to a transient electric field *E*(τ) between the probe tips. A sketch of this measurement scheme is shown in Figure 1a. In order to evenly illuminate the rf-probe contacts (total width 250 μm) the 1/*e* ^2^ diameter of the pump beam was adjusted to be 500 μm.

**Figure 1:**
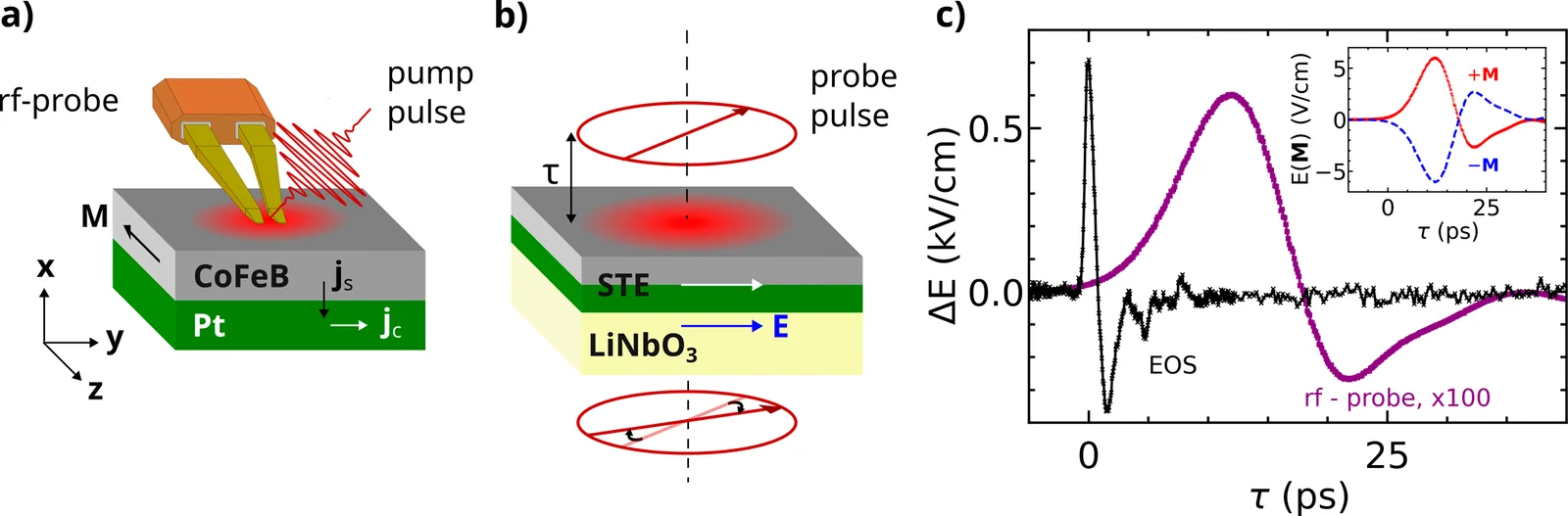
a) The pump pulse induced THz charge current **j**_c_ is detected using a rf-probe connected to a 50 GHz sampling oscilloscope. b) The electric near field **E** of **j**_c_ is probed inside the LiNbO_3_ utilizing EOS as **E** rotates the polarization (red arrow) of the probe pulse depending on the time delay τ between pump and probe pulse. c) Ultrafast response *E*(**M**, τ) of a single Pt | CoFeB layer observed using the rf-probe for two opposite magnetization directions ± **M** of CoFeB. To extract the ISHE signal the difference of both traces Δ*E*(τ) is evaluated. The trace obtained with the rf-probe (purple) is scaled by 100 to be comparable with the ultrafast response obtained by EOS (black). Both traces are obtained for Φ ≈ 0.5 mJ cm^–2^.

The optically induced current pulses originating from the ISHE are expected to scale with the magnetization of the FM layer **M** as we have **j**_c_ = θ_ISHE_ **j**_s_ × **M**/|**M**| where θ_ISHE_ is the inverse spin Hall angle.^11^ This behavior (signals odd in magnetization) is observed for all samples studied here. In the following we will consider the normalized difference signal Δ*E*(τ) = [*E*(**M**, τ) – *E*(−**M**, τ)]/2 as illustrated in Figure 1c. In our experiment **M** is oriented by an in-plane external magnetic field $B_{ext}={\left(0,0,B_{z}\right)}^{T}$ .

The signals we observe are characterized by a positive peak followed by a dip with an opposite sign. The peak width is dominated by the time resolution of the oscilloscope (10 ps). The bipolar double structure is a consequence of (1) a partial reflection of the THz pulse from the bottom surface of the substrate, and, (2) the spin current that is generated due to remagnetization of the magnetic layer.

Previously, STEs were employed to understand the spin-current propagation across interfaces.^36^ Here we aim to increase the THz signal by stacking several STEs. However, in this case all additionally created charge currents interfere destructively, as illustrated in Figure 2a. As shown in^36^ ultrathin MgO interlayers are sufficient to block spin current propagation. Thus, inserting 0.7 nm MgO between stacked STEs leads to constructive interference of all charge currents, as indicated in Figure 2b. Here, the chosen thickness is a thin as possible and as thick as needed to prevent any spin transport.^36^

**Figure 2:**
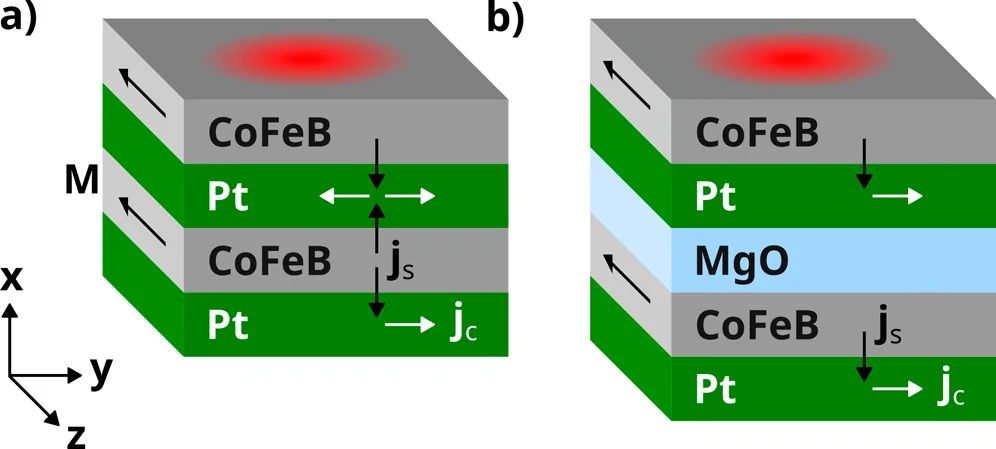
a) Laser-induced spin-current emission **j**_s_ from a CoFeB layer (gray) with magnetization **M** into an adjacent Pt layer (green). The inverse spin Hall effect converts this spin current to a transverse charge current **j**_c_ (white). In multiple Pt | CoFeB bilayers the ISHE charge currents partially cancel. b) Insertion of an insulating interlayer (e.g., MgO) between Pt | CoFeB bilayers allows the ISHE currents from multiple bilayers to be summed up.

In order to test this concept we fabricate multilayer spintronic emitters consisting of multiple HM | FM metallic bilayers stacked on top of each other, separated by an ultrathin insulating barrier, similar to.^40^ The HM layers consist of 2 nm Pt, the FM layers of 1.5 nm CoFeB, and the insulating barriers of 0.7 nm thick MgO. Top and bottom of each multilayer emitter are capped by a 1.5 nm thick TaN layer. All materials were deposited using magnetron sputtering in an ultrahigh vacuum deposition system on Al_2_O_3_ and xy-cut LiNbO_3_ substrates. A sketch of the resulting structures is shown in Figure 2b. In the following, we will study the effect of stacked bilayer STEs on the emitted THz amplitude.

We consider the THz current amplitudes for a series of *N* HM | FM bilayer stacks with MgO interlayers where *N* stands for the number of repeated Pt |CoFeB bilayers of the form *N* × (Pt | CoFeB | MgO). Here, all stacks are grown on Al_2_O_3_ (0001) substrates. In Figure 3 we plot the transient waveforms obtained for the different multilayer samples N = 1 to N = 5 using the sampling oscilloscope, as illustrated in Figure 1a. Note that each of the signal traces was recorded at the saturation pump fluence Φ_sat_, c.f. Figure 4a, which aims to mitigate the intensity losses across the multistack due to the finite penetration depth of the pump light and thus to ensure comparable demagnetized FM layers. Transmission experiments (Figure S2) reveal a 1/e penetration depth of *d*_0_ ∼ 9 nm. We find that the maximum signal amplitude at Φ_sat_ monotonically increases by a factor of ≃ 3 from *N* = 1 to *N* = 5. Qualitatively, these increased maximum amplitudes for larger *N* are in line with our intended design of the multistack where the induced charge currents add constructively. Quantitatively, however, the smaller increments for *N* > 2, even at saturation fluence, do not follow the simple expectation of a linear increase. This deviation may be explained as follows. In particular, the changing impedance with increasing thickness, which may affect coupling into the sampling tip, was not considered. Now, we account for this effect, by the following model: The total current *I*_N_ flowing inside the waveguide is the sum of the individual currents *I*_0_ inside a bilayer multiplied by the transmission coefficient 1 – Γ_N_ from the STE into the medium where it is detected. This accounts for the thickness (*N* dependent) absorption for each sample. Hence, the dependence of the field Δ*E*_N_ caused by *I*_N_ on the laser fluence Φ can be written as follows:

$$\Delta E_{N}=\{\begin{array}{ll} \left(1-\Gamma_{N}\right)\overset{N-1}{\underset{n=0}{\sum }}\left(\eta \Delta E_{0}\Phi \exp\left(-n/N_{0}\right)\right), & \Phi <\Phi_{sat}\\ (1-\Gamma_{N})\;N\Delta E_{0}, & \Phi \geq \Phi_{sat} \end{array}$$ 1

Here ηΔ*E*_0_ is the conversion ratio from the optical pump fluence to the THz amplitude, Γ_N_ = |*R*_STE_ – *R*_M_|/(*R*_STE_ + *R*_M_) is the standing wave ratio involving the resistance of the multilayered STE *R*_STE_, the resistance of the medium of detection *R*_M_, and the optical 1/e decay length of our HM | FM stacks, *N*_0_ = 2.4. *R*_STE_ can be estimated using the DC sheet resistance, *R*_sheet_, determined via the Van der Pauw method (see Figure S3) and shows a hyperbolic decay with *N*. *R*_sheet_ is weighted with a geometrical factor α to convert the sheet resistance into the effective resistance of our stacks. When assuming a cuboid α is the conductor length divided by its width. As the geometry for the rf-tip (Figure 1a) and EOS (Figure 1b) does not allow a direct calculation, the value of α is found by fitting instead, c.f. Figure 4b,d. The influence of changing α can be comprehended using the provided script on Zenodo.^41^ The sum in eq 1 shows a monotonic increase with *N*, while 1 – Γ_N_ is decreasing for (large) *N*. Utilizing eq 1 to fit our data leads to a reasonable agreement between the experimental result and our model, as shown in Figure 4b. This result shows that the observed behavior can be well described when optical absorption and impedance mismatch for the THz waves are considered.

**Figure 3:**
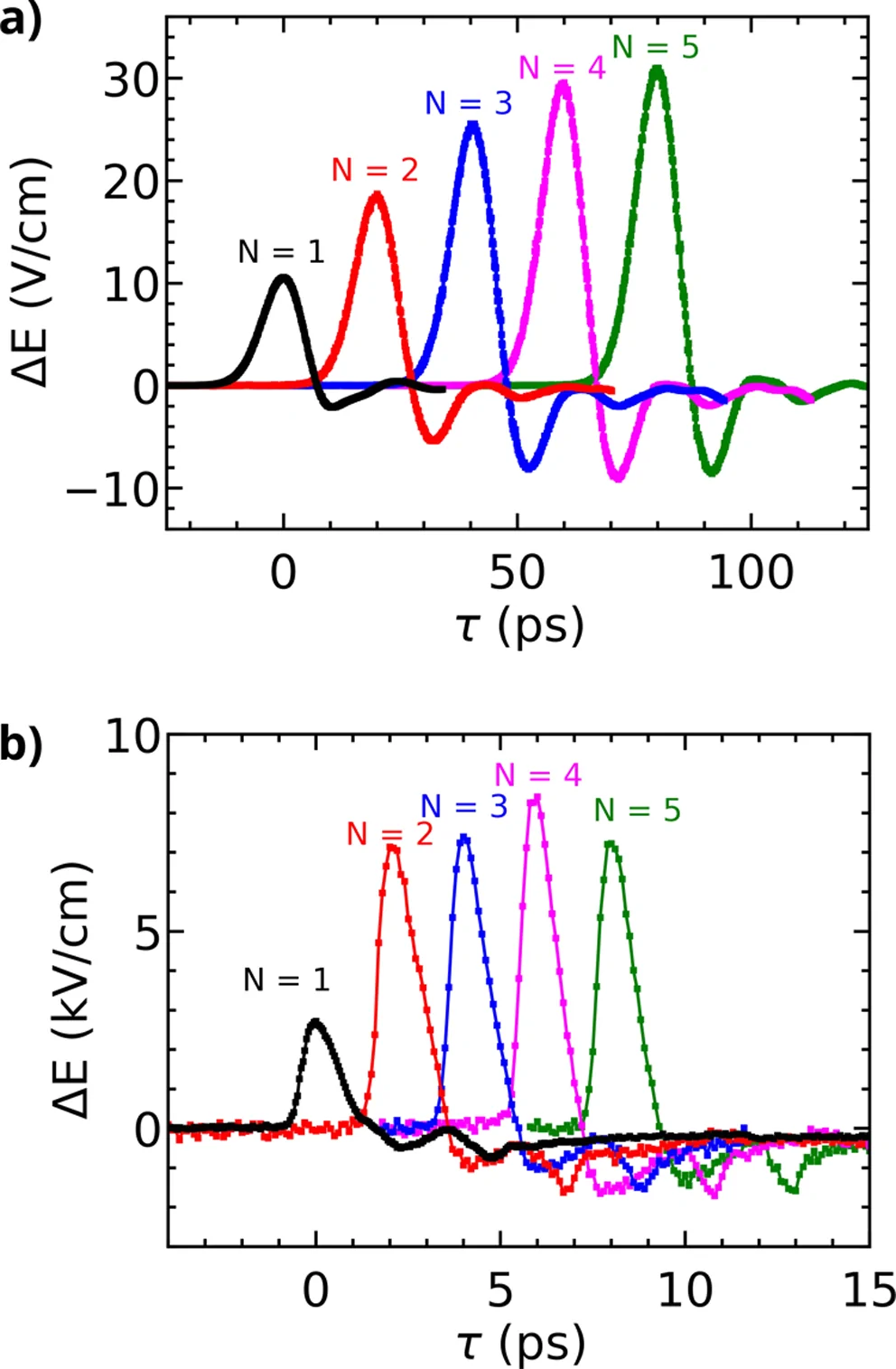
Time-resolved ISHE response, Δ*E*, generated by optical pumping *N* stacked bilayers which is either detected by a) a fast sampling oscilloscope combined with a rf-tip (see Figure 1a) or by b) employing EOS and optically probing on the emitter (see Figure 1b). Each trace is obtained at saturation fluence for the respective *N* as seen in Figure 4a) and c). For better visibility each trace is shifted to the previous one by a) 20 ps and b) 2 ps in time (the *N* = 1 data are not shifted).

**Figure 4:**
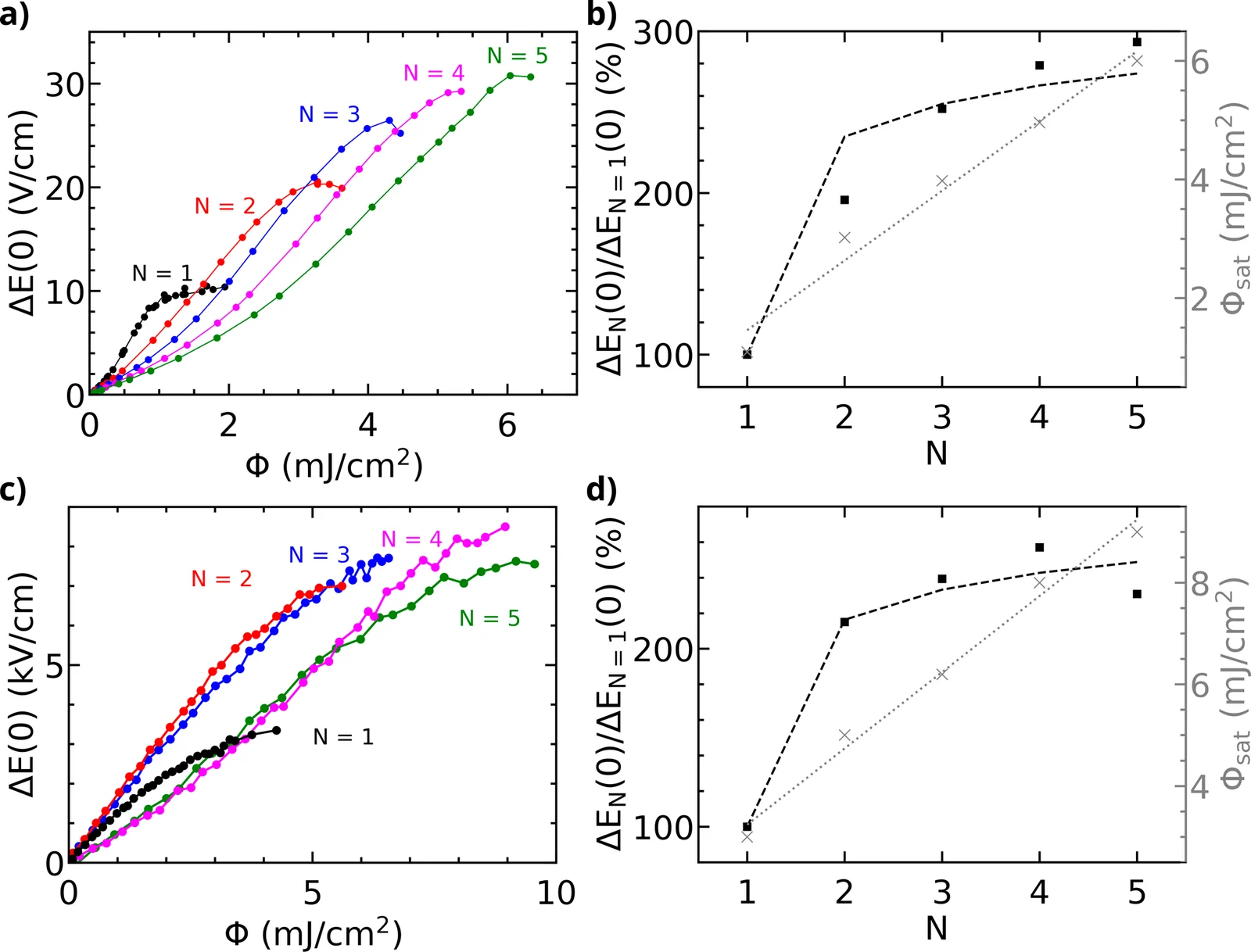
Amplitude Δ*E*(τ = 0) for *N* = 1 to 5 with increasing fluence Φ obtained using either a) the rf-probe or c) EOS and optically probing on the emitter. b,d) Amplitudes Δ*E*_N_(0) (black) of a,c) measured at saturation fluence Φ_sat_ (gray) for each *N* normalized to Δ*E*_*N*=1_(0). The black dashed line shows a fit of Δ*E*_N_(0)/Δ*E*_*N*=1_(0) using eq 1 with b) α = 0.38 and the tip resistance *R*_M_ = 50 Ω or d) α = 0.4 and the wave impedance of LiNbO_3_ at 1 THz *R*_M_ = 56 Ω.^1^ Additionally, a linear relation is fit to Φ_sat_ as guide for the eye (dotted gray line).

At Φ < Φ_sat_ a monotonic increase of Δ*E* is observed for all samples, as illustrated in Figure 4a. Yet, this increase scales nonlinearly even at small fluences. In particular, the magnitude shows a less steep increase at lower fluences compared to larger fluences. This nonlinear behavior is a consequence of the very limited detection bandwidth of the oscilloscope in combination with a physical process that generates more low frequency signal components at larger fluence. Specifically, one can expect that the remagnetization dynamics slows down for larger fluences.^42,43^ In the following, we corroborate this idea by measuring the ultrafast photocurrents with a THz bandwidth.

In general, the expected shape of Δ*I*(τ) should be proportional to the injected *j*_s_ and consequently to the demagnetization $\frac{\partial }{\partial t}M$ .^22^ For extremely short pump pulses (10 fs) the demagnetization occurs, for comparable samples to those of the present work, on a time scale of about 50 fs,^12,16^ while the remagnetization time strongly depends on the pump fluence and occurs on a time scale of a few hundred fs for low fluences and up to ns for large fluences.^42,43^

To obtain a THz bandwidth for the current detection we use an all-optical pump–probe method. Specifically, an electro-optic sampling (EOS) scheme is implemented, as illustrated in Figure 1b). For this purpose, the samples are fabricated on piezolectric LiNbO_3_ substrates, as LiNbO_3_ exhibits a strong Pockels effect, making it therefore an efficient electric near-field detector. Although, it shows strong absorption above 4 THz^44–46^ this is of no concern for our EOS detection scheme, as our 300 fs laser pulses limit the achievable emitter bandwidth to 3 THz. In this scheme, a linearly polarized fs probe pulse is used to measure the transient THz-electric field in the LiNbO_3_ substrate beneath the HM | FM bilayer where the polarization of the probe pulse is rotated due to the Pockels effect. Using a balanced photo detector this effect is probed at a given time delay τ between the pump and probe pulses. A detailed description of the EOS setup is provided in Section S4. The 1/*e* ^2^ diameters for the pump and probe pulses are 300 μm and 60 μm, respectively. A typical measurement is shown in Figure 1c. We assign the positive peak in Δ*E* to the ultrafast demagnetization and the subsequent negative peak to the slower remagnetization of the CoFeB layer. The additional negative peak at τ ∼ 5 ps is attributed to the reflection of the THz wave at the backside of the substrate. This is explained in detail in Figure S4. In total a bandwidth of 1 THz is achieved, as can be seen in Figure S1. This is lower than expected for a prototypical STE^11^ but can be explained by the time resolution of our EOS experiments which is limited by two factors: (1) the 300 fs full width half-maximum (fwhm) pulse duration of the amplified laser system (pump and probe pulses) and (2) the copropagation between THz and optical probe pulse inside the LiNbO_3_ substrate (as illustrated in Figure S5). The latter effect limits the EOS bandwidth to about 1 THz.

Repeating the fluence dependence of Δ*E* for *N* = 1 with EOS shows that the remagnetization dynamics strongly depend on the chosen pump fluence (as shown in Figure S6 to Figure S8). Finally, the THz peak amplitude as a function of the pump fluence scales linearly, as expected. We would like to point out that our results also allow us to reproduce the nonlinear fluence scaling for the low-frequency tail of the THz EOS data as illustrated in Figure S9.

We repeat the fluence dependence of Δ*E* utilizing EOS for *N* > 1. As seen in Figure 3b for Φ = Φ_sat_ a monotonous increase is again observed, although the maximum increase of ∼2.5 and the individual values for each *N* differ as compared to Figure 4b. We attribute this to the different electromagnetic environment and, thus, the impedance of the films grown on LiNbO_3_. Using eq 1 to fit the data, as depicted in Figure 4d, acceptable agreement is found up to *N* = 4. The fluence dependence of Δ*E* for each *N* (see Figure 4c) shows a linear increase, which flattens when approaching saturation up to *N* = 3. For *N* = 4 and *N* = 5 a nonlinear behavior is found for small Φ. This is most likely caused by the uneven demagnetization of the different FM layers (finite optical penetration), leading to only the upper bilayers to effectively contribute to the total THz field, while the reduced impedance quenches the coupling into the LiNbO_3_.

In total, investigating the multistack STEs with sub ps time resolution shows the expected increase in THz amplitude for all fluences. Within the used fluences (c.f. Figure 4a,c and Figure S10a) our samples exhibit a reproducible behavior. For *N* = 1 a damage threshold of ∼6.5 mJ cm^–2^ is observed which is indicated by reduced reflectivity, changes in topography and irreversible magnetic properties.

Lastly, we study our multistacks as a possible on-chip THz current source. Thus, we embed the STEs into Au waveguide structures and investigate the coupling of the THz current into and its propagation along the waveguide. A sketch of the design is shown in Figure 5, where the emitter is fabricated as a *w*_emitter_ = 250 μm wide stripe and is incorporated in a 140 nm × 150 μm × 3 mm coplanar stripline with a 60 μm gap. First, the fluence dependence when coupling into the waveguide is investigated for all *N* by positioning the probe beam right next to the emitter (*z* ∼ 160 μm, as illustrated in the inset of Figure 6a). As seen in Figure S10 we find a good agreement between the experiments and our model. Second, we look at the propagation of the THz wave inside the waveguide by scanning the probe beam along the *z*-direction and detecting the transient electric field, Δ*E*. The results for *N* = 3 in Figure 6a can be divided into two parts, (1) the coupling of the THz current into the waveguide and (2) the propagation along the waveguide. When comparing Δ*E* before (*z* = 0) and after (*z* = 200 μm) coupling into the waveguide we see a drop in amplitude by about a factor of 2 and a broadening in time of the pulse (compare with the FFT in Figure 6b as well). The former can be explained by the pulse splitting up into the ±*z*-directions of the waveguide. The latter is caused by the runtime delay between generation of the THz pulse along the emitter and its arrival/detection in the waveguide. In a simple picture this behavior can be understood as a set of delta like pulses generated at different positions |*z*_*i*_| ≤ *w*_emitter_/2, for a given emitter width *w*_emitter_ along the *z*-direction, which propagate with their group velocity *v*_g_ along the waveguide. Detecting this pulse train at a position *z*_0_ > *w*_emitter_/2 yields a waveform with a finite temporal width of *w*_emitter_/*v*_g_ ≤ *w*_emitter_/*c* ≃ 0.8 ps in strong contrast to the original delta like pulses. Using the data in Figure 6a gives an average *v*_g_ = 0.22 *c* which is in good agreement with the phonon polariton dispersion along the *z*-direction (extra ordinary optical axis) in LiNbO_3_.^47^ Applying a more accurate description, including the intensity profile of the pump laser and the finite temporal width of the initial THz pulses, gives a very good agreement between model and experiment, as discussed in Section S9. Studying Figure 6a further shows that over a distance of 600 μm Δ*E* becomes bipolar and loses ∼50% amplitude. This can be seen from a decreasing bandwidth and magnitude as well as a developing peak around ∼100 GHz in the corresponding FFTs in Figure 6b. Utilizing the propagation data allows us to obtain the dispersion relation *f*(*k*) for wavevectors *k* = *k*_*z*_ either by a direct 2D-FFT (see Figure S12) or by calculation from the complex Fourier coefficients, as described in Section S10 and as can be seen in Figure S13. Both methods yield a dispersion which agrees well with the literature^47^ but is nonlinear. Thus, with increasing *f* the ratio between *v*_g_ and phase velocity *v*_p_ gets smaller than unity leading to an increased dispersion for the high frequency components of the THz pulses. To summarize, our data reveal the dispersive nature of the waveguide and indicates the need to specially design the waveguide structure to support a linear dispersion up to THz frequencies.

**Figure 5:**
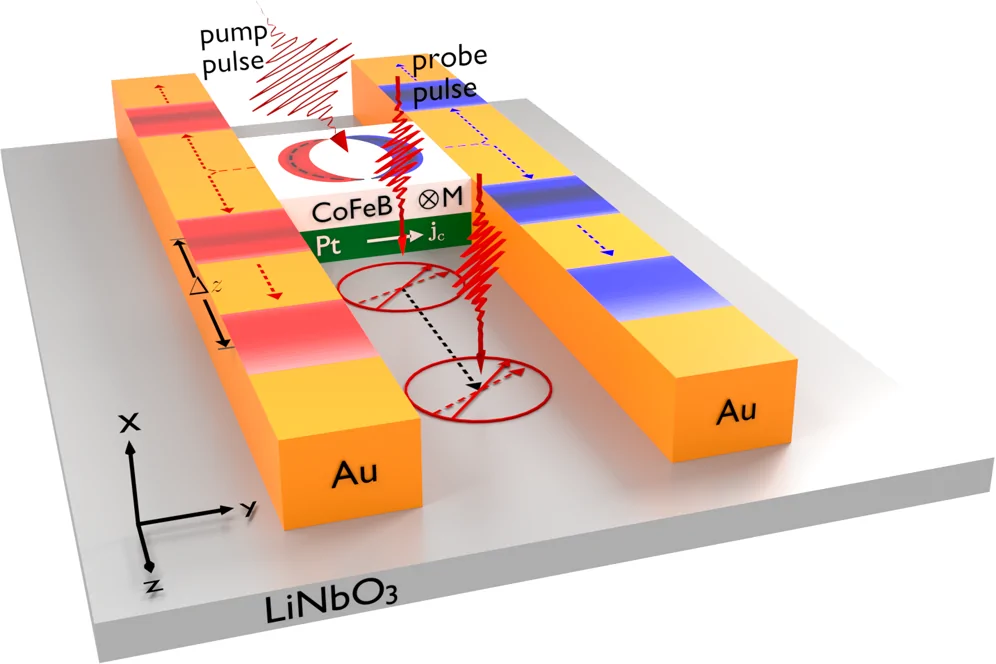
Geometry of the on-chip electro-optic sampling experiments: The spintronic emitter terminates a waveguide structure. After coupling into the waveguide, the laser-induced current pulse **j**_c_ branches into a positive (red) and negative (blue) part along the ±*z*-direction respectively, creating a propagating, transient electric field **E**(*z*, τ) across the gap. By moving the probe beam inside the gap along the *z*-direction (black dashed line), this propagation is mapped.

**Figure 6:**
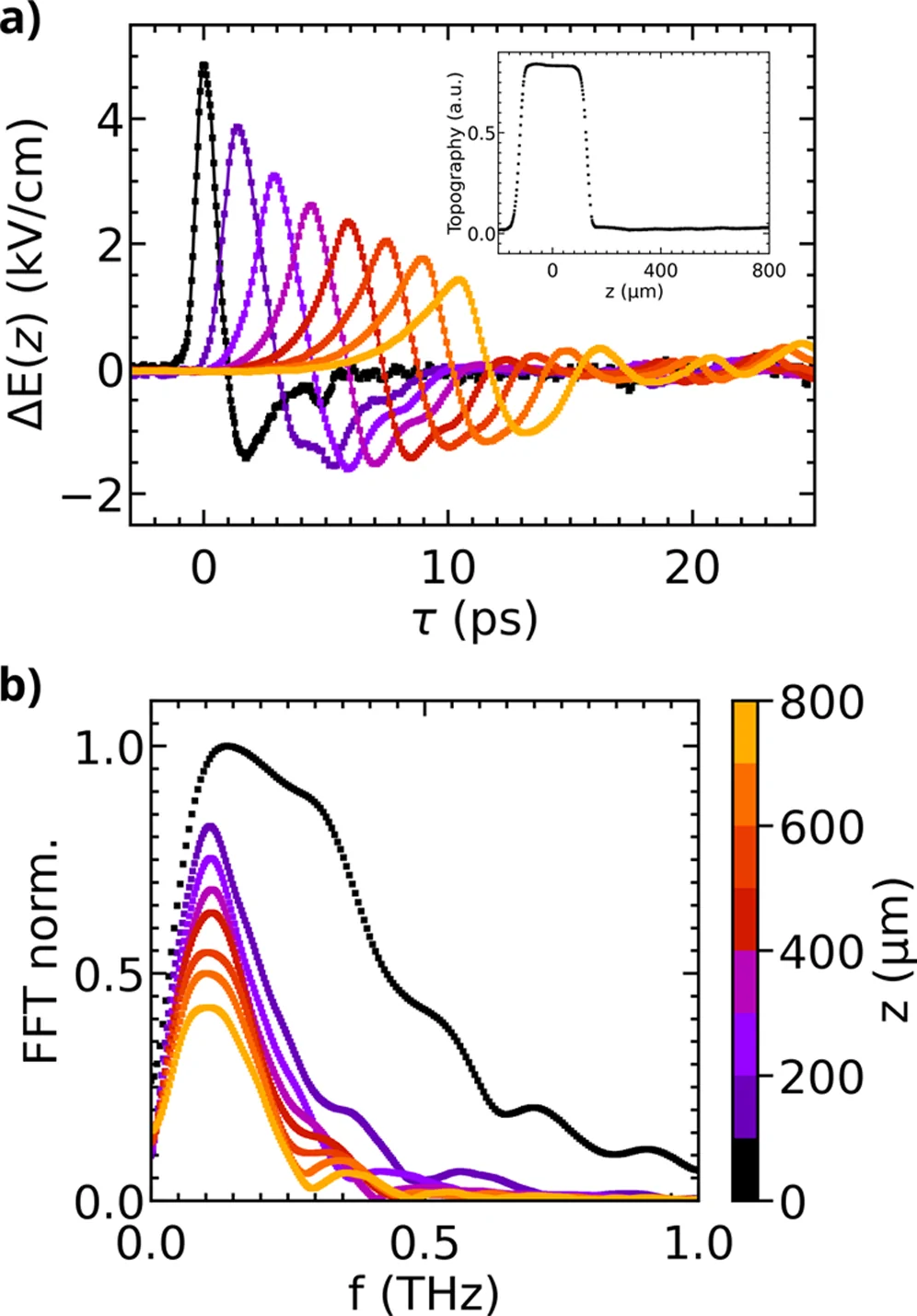
a) Propagation of the THz pulse inside the waveguide for *N* = 3 at Φ ≈ 2 mJ cm^–2^. A full waveform Δ*E*(*z*) is obtained for different positions *z* of the probe beam inside the gap starting on the STE (*z* = 0) and moving in positive *z*-direction as sketched by the dashed black line in Figure 5. As depicted by the topography in the inset the STE is situated between *z* = ±125 μm. For *z* > 125 μm all waveforms are multiplied by two to account for the branching of the pulse at *z* = 0 in the ±*z*-direction. b) FFT of each waveform in a) normalized to the maximum of the FFT at *z* = 0.

In conclusion, we have demonstrated that multilayer spintronic THz emitters based on repeated Pt | CoFeB bilayers separated by ultrathin MgO barriers provide a promising pathway toward enhanced THz current generation. By suppressing spin-current propagation between adjacent bilayers while preserving constructive addition of the generated charge currents, the multilayered device enables a substantial increase in the emitted THz signal compared to conventional single-bilayer emitters. Both GHz-bandwidth electrical sampling and THz-bandwidth electro-optic sampling reveal an enhancement of the transient THz amplitude with increasing number of bilayers, reaching an enhancement factor of up to ∼3 for five stacked emitters. In the measurements with rf-probe tip (50 GHz bandwidth) this seems to result in a decreased emitter efficiency, i.e. smaller amplitudes observed at the same optical pump fluence. However, it should be noted that a quantitative analysis is reliable only when using EOS at THz bandwidth, as it largely avoids time averaging and filtering effects. EOS reveals an increased emitter efficiency of 1.5 up to *N* = 3.

We further show that the observed scaling behavior can be quantitatively described by a model incorporating optical absorption and impedance matching between the multilayer stack and the detection environment. Time-resolved electro-optic measurements confirm that the intrinsic ultrafast spintronic THz generation process also occurs in a multilayer geometry and we clarify the origin of the nonlinear fluence dependence observed in bandwidth-limited electrical measurements.

Finally, by integrating the multilayer emitters into coplanar waveguide structures, we demonstrate on-chip generation and propagation of ultrafast THz current pulses. The measured propagation dynamics reveal strong dispersive effects originating from the phonon polariton response of the LiNbO_3_ substrate, emphasizing the importance of dispersion engineered waveguide concepts for future integrated THz circuitry.

Our near field experiments complement previous work which addressed the THz emission only in the far field.^39,40^ Hence, our results establish multilayer STEs as a scalable platform for increasing THz current amplitudes without sacrificing ultrafast operation. Beyond broadband THz spectroscopy, these multilayer STEs therefore represent a promising route toward compact, high-amplitude, on-chip THz sources for ultrafast electronics, spintronics, and integrated THz communication technologies.

## Supplementary Material



## Data Availability

The data that support the findings of this study are available from the following data repository DOI: 10.5281/zenodo.22098749.

## Notes

FFT of Figure 1, the transmission values for the fabricated samples as well as their sheet resistance, a detailed description of the EOS detection scheme, the influence of the fluence on the frequency components of the THz pulse, the fluence dependence for the on-chip EOS approach, calculations on the run time delay broadening and the dispersion relation of our waveguide (PDF)
