# Supplementary material for: Multilayer Spintronic THz Emitters for On-Chip Ultrafast Current Generation
Source: Nano Lett. 2026 Oct 22;26(43):14837–44. doi: 10.1021/acs.nanolett.6c02697 (PMC13741912; doi:10.1021/acs.nanolett.6c02697)
Supplement: Supplementary file 1 [file nl6c02697_si_001.pdf]

Supporting Information: Multilayer Spintronic THz Emitters for On-Chip Ultrafast Current Generation

Wolfgang Hoppe,[†] Mohamed Amine Wahada,[‡] Paul Marschall,[†] Stuart S. P. Parkin,[‡,¶] and Georg Woltersdorf[*,†,‡,¶]

† *Institute of Physics, Martin Luther University Halle-Wittenberg, von Danckelmann Platz 3, 06120 Halle, Germany*

‡ *Max Planck Institute for Microstructure Physics, Weinberg 2 06120 Halle, Germany*

¶*Cluster of Excellence - Center for Chiral Electronics, Halle-Berlin-Regensburg, Germany*

E-mail: georg.woltersdorf@physik.uni-halle.de

S1 FFT: rf-probe and EOS

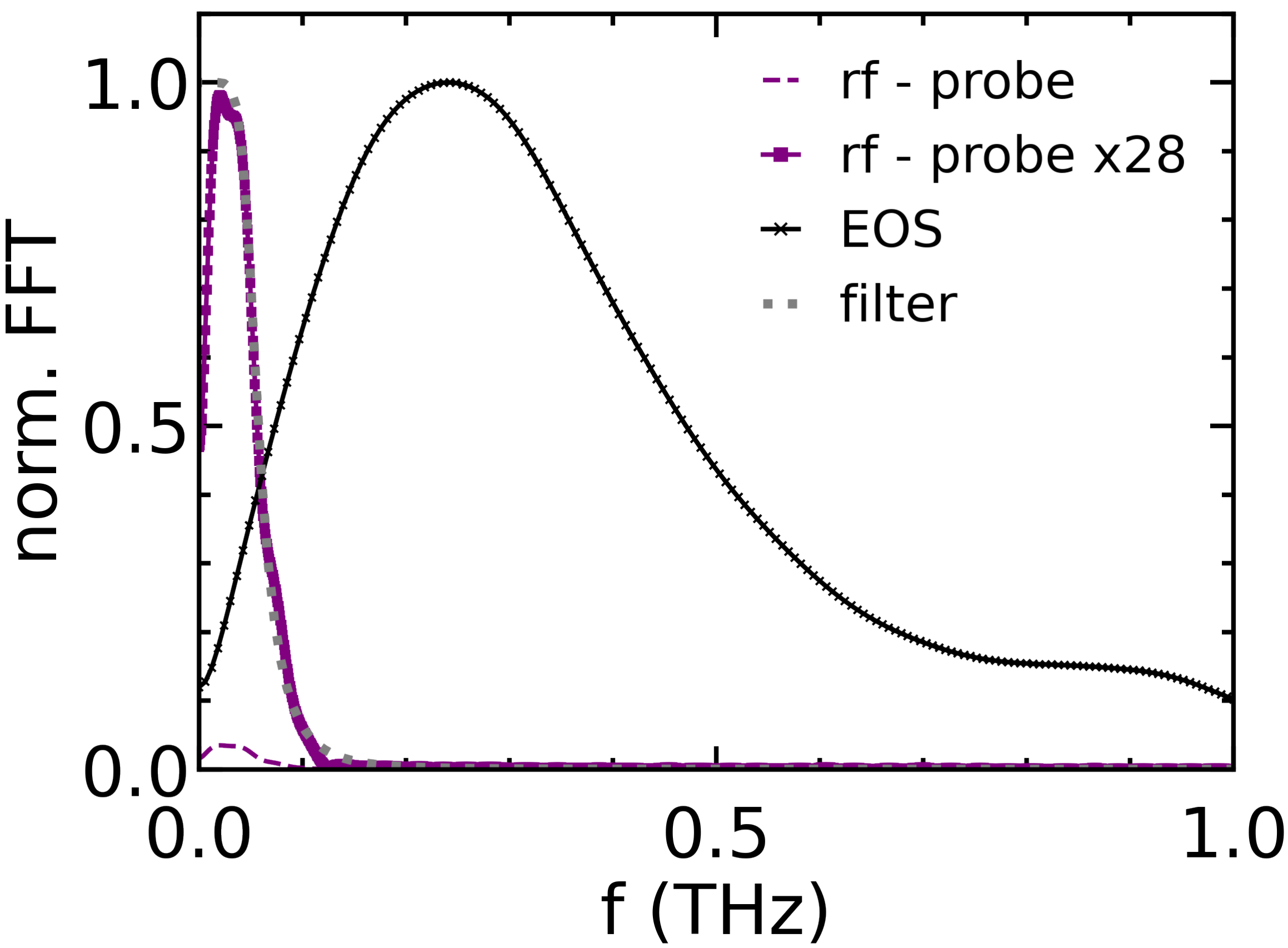

Figure S1: FFT magnitude of the rf-tip waveform (dashed purple) and EOS waveform (black x) shown in **??**c normalized to the maximum value of EOS FFT. Additionally, a fourth order, 50 GHz Butterworth filter (dashed grey) is plotted, representing the filter function of the oscilloscope. Comparing it with the scaled FFT of the rf-tip waveform (purple square) shows excellent agreement. For the FFT of the EOS data only data points until the second zero crossing are included to exclude the backreflection of the THz wave at $\tau = 5\,\mathrm{ps}$. Note, that we define the bandwidth as the frequency value where the norm. FFT magnitude drops to 10 %.

S2 Transmission

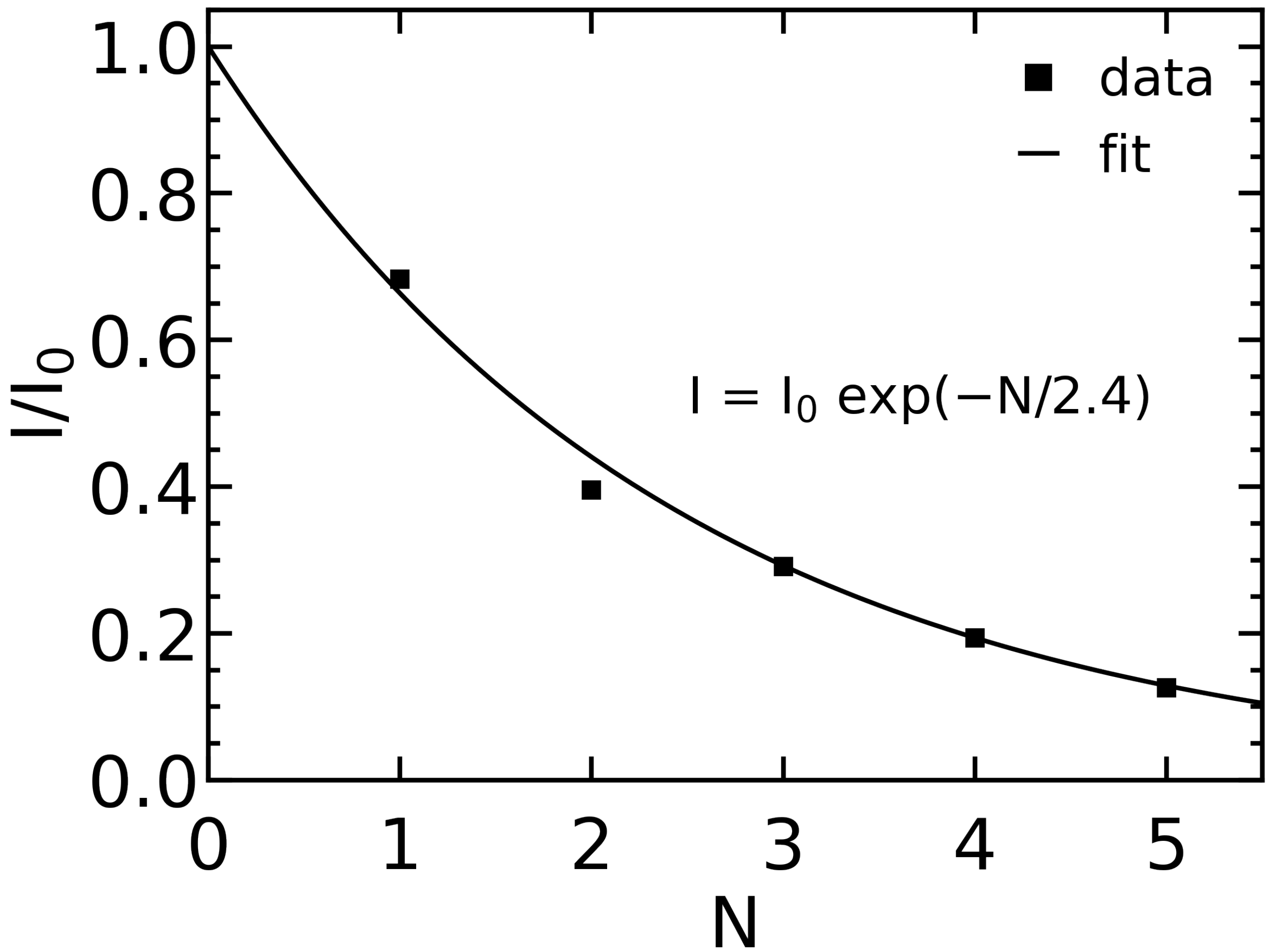

Figure S2: Transmission I/I_0 of the infrared probe pulse through the STE for different numbers of bilayer stacks N. The reduction in intensity due to the $LiNbO_3$ substrate is already accounted for. An exponential function was fit to the data yielding a decay constant of 2.4 stacks or 8.5 nm of metal.

S3 Sheet resistance

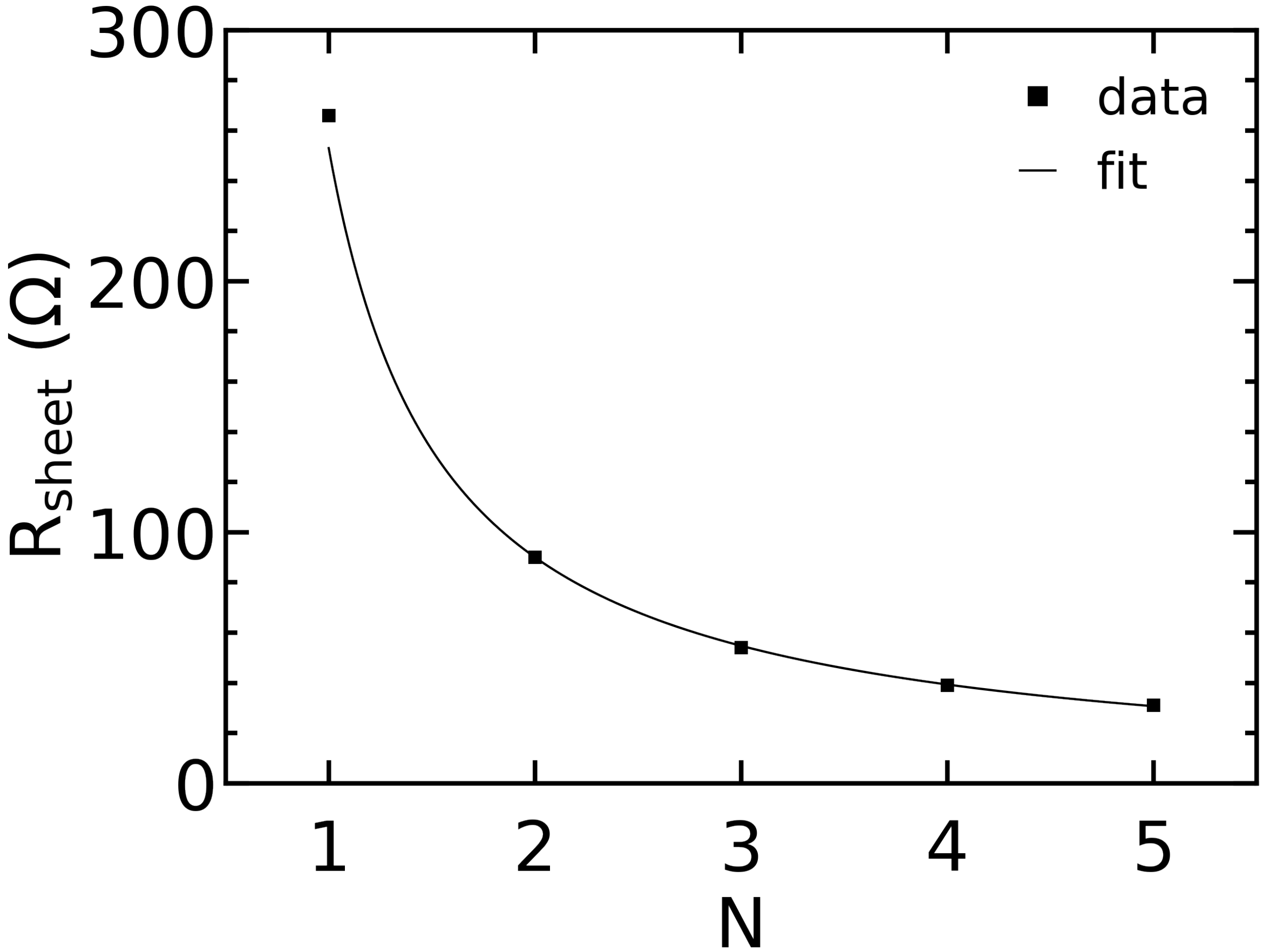

Figure S3: The sheet resistance R_{sheet} of the multi stacks grown on Al_2O_3 obtained by employing the van der Pauw Method. A hyperbolic dependence $R_{\text{sheet}} = 140\,\Omega/(N-0.44)$ is fit to the data.

S4 Electro-optic sampling

To evaluate the effect of an electric field $\boldsymbol{E}$ inside the $LiNbO_3$ on the probe pulse polarisation we first need to calculate the change of the refractive index ellipsoid $\Delta\left(\frac{1}{n_i^2}\right)$ due to the Pockels effect:

$$\Delta\left(\frac{1}{n_i^2}\right) = \sum_{j=1}^{N=3} r_{ij} E_j, \quad \text{for} \quad 1 \leq i \leq 6 \tag{1}$$

As $LiNbO_3$ belongs to the $3m$ point group[S1] its electro-optic tensor r_{ij} can be written as :

$$r_{ij} = \begin{pmatrix} 0 & 0 & 0 & 0 & 0 & r_{42} \\ -r_{22} & r_{22} & 0 & 0 & r_{42} & 0 \\ r_{13} & r_{13} & r_{33} & 0 & 0 & 0 \end{pmatrix}^T \tag{2}$$

With $\boldsymbol{E} = (0, E_\mathrm{y}, 0)^T$ and an propagation of our probe pulse in x-direction the final index ellipse simplifies to:

$$\frac{1}{n_\mathrm{y}^2}\,y^2 + \frac{1}{n_\mathrm{eo}}\,z^2 + 2r_{42}E_\mathrm{y}\,yz = 1, \qquad n_\mathrm{y} = \frac{n_\mathrm{o}}{\sqrt{1 - n_\mathrm{o}^2 r_{22} E_\mathrm{y}}} \tag{3}$$

Here n_o and n_ao are the ordinary and extraordinary refractive indices of $LiNbO_3$ along. When further assuming $2r_{42}E_\mathrm{y} \ll 1$ and $n_\mathrm{o}^2 r_{22} E_\mathrm{y} \ll 1$ the yz term in Eq. (3) can be neglected and the n_y can be approximated using a Taylor expansion around $E_\mathrm{y} = 0$, leading to a new refractive index in y-direction $n_\mathrm{y} \approx n_\mathrm{o}(1 + \frac{1}{2} n_\mathrm{o}^2 r_{22} E_\mathrm{y})$. This allows to calculate the additional phase delay $\Delta\phi$ caused by $\boldsymbol{E}$ between the y- and z-component of the probe pulse polarisation to $\Delta\phi = \frac{\pi d_\mathrm{eff}}{\lambda} n_\mathrm{o}^3 r_{22} E_\mathrm{y}$. Here λ refers to the wavelength of the probe pulse and d_eff to the thickness of the $LiNbO_3$ substrate effectively penetrated by E_y.

To predict the observable effect of this additional $\Delta\phi_\mathrm{E}$ it is beneficial to translate the detection scheme into the Jones formalism. After the polarisation of the probe pulse is set to $\boldsymbol{E}_\mathrm{init} = (\alpha, \beta)^T$ it passes the first $LiNbO_3$ substrate with the waveguide on top, then a second $LiNbO_3$ plate of the same thickness d but rotated by the angle χ close to 90° and then a combination of $\lambda/2$-waveplate and Wollaston prism. Therefore the electric field $\boldsymbol{E}_\mathrm{final} = (E_\parallel, E_\perp)^T$ after the second $LiNbO_3$ substrate is calculated by:

$$\begin{gathered} \boldsymbol{E}_\mathrm{final} = \underline{\boldsymbol{R}}(-\chi)\underline{\boldsymbol{L}}\,\underline{\boldsymbol{R}}(\chi)\,\underline{\boldsymbol{L}}_\mathrm{E}\boldsymbol{E}_\mathrm{init}, \\ \underline{\boldsymbol{R}}(\chi) = \begin{pmatrix} \cos\chi & -\sin\chi \\ \sin\chi & \cos\chi \end{pmatrix}, \quad \underline{\boldsymbol{L}} = \begin{pmatrix} e^{-i\delta} & 0 \\ 0 & e^{i\delta} \end{pmatrix}, \quad \underline{\boldsymbol{L}}_\mathrm{E} = \underline{\boldsymbol{L}} \begin{pmatrix} e^{i\Delta\phi} & 0 \\ 0 & e^{i\Delta\phi} \end{pmatrix} \end{gathered} \tag{4}$$

Here $\underline{\boldsymbol{L}}$, $\underline{\boldsymbol{L}}_{\mathrm{E}}$ are the Jones matrices for the $LiNbO_3$ substrates with and without external field, $\delta = \frac{\pi d}{\lambda}(n_{\mathrm{eo}} - n_{\mathrm{o}})$ the phase shift caused by the field independent birefringence of $LiNbO_3$ and $\underline{\boldsymbol{R}}(\chi)$ the rotation matrix around χ. The Wollaston prism then splits $\boldsymbol{E}_{\mathrm{final}}$ in its orthogonal components, the respective intensities each detected at a photodiode as a voltage $U_{\parallel} \propto E_{\parallel} E_{\parallel}^*$ and $U_{\perp} \propto E_{\perp} E_{\perp}^*$. The asterisk denotes the complex conjugate. At the lock-in amplifier the difference of both voltages $\Delta U = U_{\perp} - U_{\parallel}$ is obtained. Evaluating Eq. (4) the following equation for ΔU can be found:

$$\begin{aligned}\Delta U/\eta_{\mathrm{PD}} = (\alpha^2 - \beta^2)(\cos^4\chi + \sin^4\chi + \frac{1}{2}\sin^2(2\chi)(\cos 2\delta - 2\sin^2\delta)) + \\ 4\alpha\beta \sin 2\chi \sin\delta \langle \cos^2\chi \sin(3\delta + \Delta\phi(E_{\mathrm{y}})) + \sin^2(\chi)\sin(\delta + \Delta\phi(E_{\mathrm{y}}))\rangle\end{aligned} \tag{5}$$

Here η_{PD} denotes the intensity to voltage conversion of the photodiode. This bulky equation can be tremendously simplified when considering the case of $\alpha = \beta = E_0/\sqrt{2}$, employing the Taylor expansion on $\sin(\delta + \Delta\phi(E_{\mathrm{y}}))$ and $\sin(3\delta + \Delta\phi(E_{\mathrm{y}}))$ for small E_{y} and using the fact, that upon modulating the magnetic field the lock-in is only sensitive to terms containing a dependence in E_{y}. Therefore the demodulated voltage difference U_{PD} simplifies to:

$$U_{\mathrm{PD}} = \Gamma(\chi)\, r_{22} E_{\mathrm{y}} \tag{6}$$

Now a linear dependence on our field inside the gap, i.e the THz field or the calibration field, is given. As the result depends on the prefactor $\Gamma(\chi)$ it is crucial that the geometry of detection is not changed in between different measurements, to ensure comparability. This is achieved by defining the exact position of each optical component as well as using calibration values for the unmodulated photovoltages. Lastly a note on the approximations used in the above calculations; the deviations between the approximated value and the "real" value deviate $\leq 5\,\%$ up to $E_{\mathrm{y}} = 1 \times 10^8\,\mathrm{V\,m^{-1}}$ using values of $r_{22} = 6\,\mathrm{pm\,V^{-1}}$ and $r_{42} = 28\,\mathrm{pm\,V^{-1}}$ for the unclamped case.[S1]

S5 $LiNbO_3$ THz wave reflection

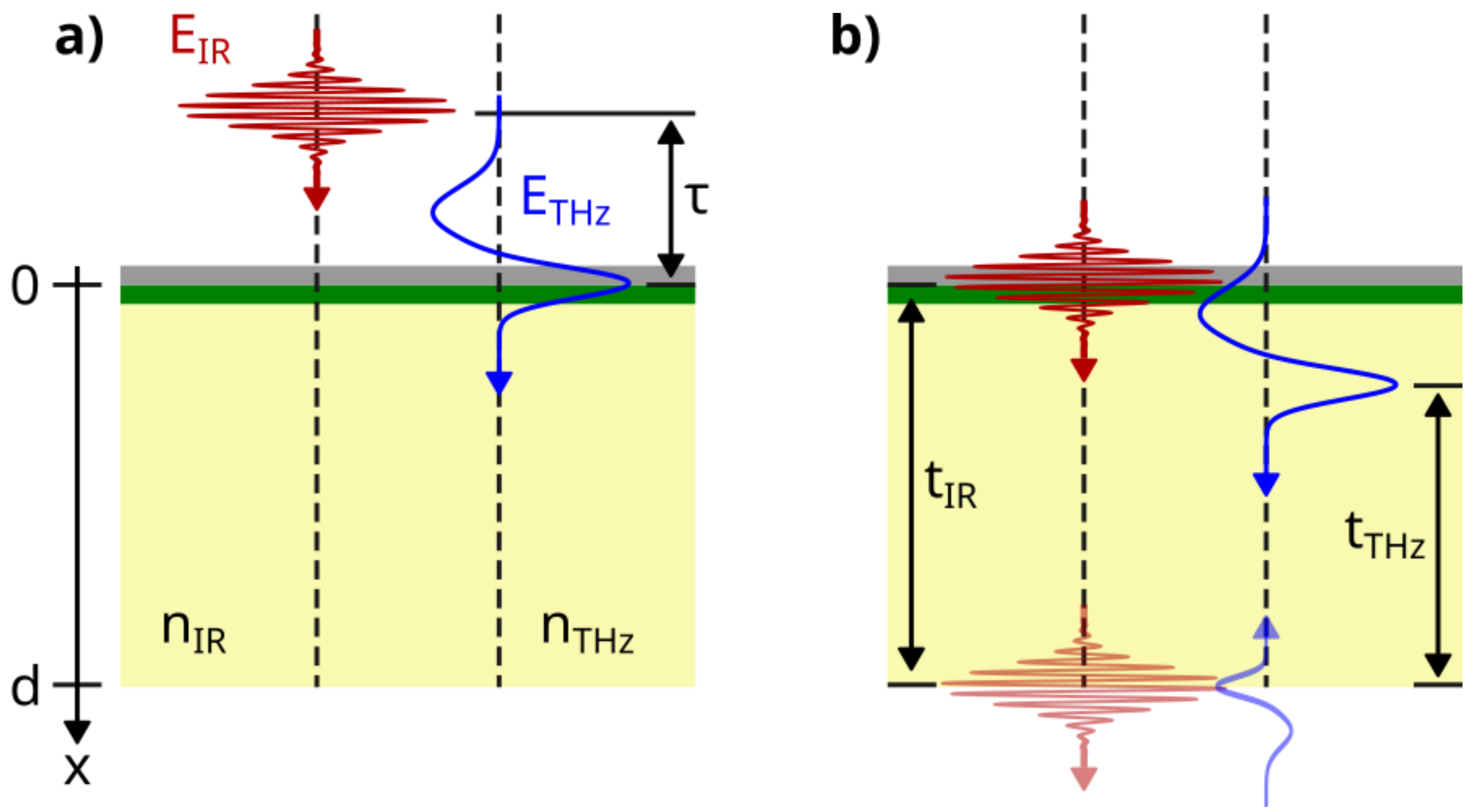

Figure S4: a) The pump pulse induces a THz wave E_{THz} inside the STE ($x = 0$) which is followed by the probe pulse E_{IR} at a given initial delay τ between them. Note, that the horizontal offset between them is just for clarity and they are indeed copropagating. b) When arriving at $x = 0$, the polarization of E_{IR} is rotated proportional to E_{THz} still inside the STE. Both waves propagate through the $LiNbO_3$ and reach the bottom side ($x = d$) at $t_{IR} = \frac{d}{c} n_{IR}$ and $t_{THz} = \frac{d}{c} n_{THz} - \tau$, with the speed of light c, the thickness of the $LiNbO_3$ slab $d = 500\,\mu m$ and the ordinary (extraordinary) refractive indices for the probe pulse $n_{IR} = 2.23$[S1] and the THz wave $n_{THz} = 6.48$.[S2,S3] If $\tau = \frac{d}{c}(n_{IR} - n_{THz}) \sim 7\,ps$ both arrive at the same time, sketched by the transparent waveforms, causing an additional rotation of the probe pulse. As the polarity of E_{THz} inverts upon reflection, this rotation is seen as a peak of opposite sign compared to $E(\tau = 0)$. We identify the negative peak at $\tau \sim 5\,ps$ in our signal ΔE (e.g. **??**b) with this backreflection. The deviation between predicted and observed τ may be explained by the assumption that the THz wave consists only of one frequency component (1 THz) but in principle has a broadband spectrum (0.1 GHz to 30 THz)[S4] and the refractive index / dielectric constant varies in this frequency range.[S5] In turn, such variations alter the expected τ.

S6 $LiNbO_3$ - time broadening

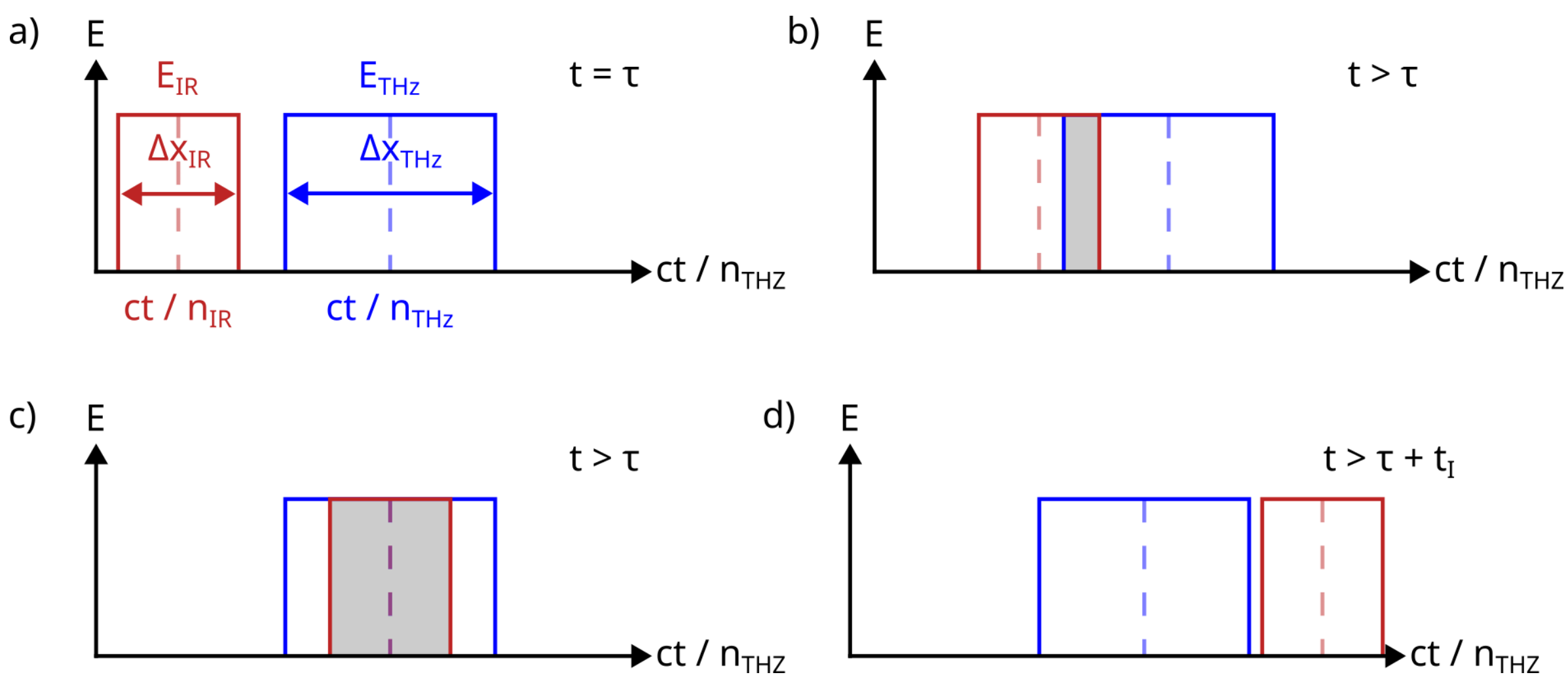

Figure S5: a) THz wave (E_{THz}) and probe pulse (E_{IR}) propagate inside the $LiNbO_3$ at a given delay τ between pump and probe. For simplicity both pulses are assumed to be of rectangular shape with respective spatial width of Δx_{IR} and Δx_{THz} moving with velocities c/n_{IR} and c/n_{THz}. b), c) Inside the $LiNbO_3$, $c/n_{\mathrm{IR}} > c/n_{\mathrm{THz}}$ and hence E_{IR} outpaces E_{THz} leading for $t > \tau$ to a finite interaction (grey shaded area) between them. d) After the interaction time t_{I} the pulses move separately again.

Besides the temporal broadening of $\Delta E(\tau)$ due to the convolution between IR probe pulse (E_{IR}) and THz pulse (E_{THz}) there is an additional contribution due to the spatial overlap of both inside the $LiNbO_3$. After the generation of E_{THz} in the STE it propagates through the $LiNbO_3$ and is followed by E_{IR}. Even if at a fixed delay τ there is no spatial overlap of both at the vicinity of the STE, there still can be some interaction that contributes to $\Delta E(\tau)$. The reason for this is that the Pockels effect occurs as long as there is spatial overlap between both pulses. This is illustrated in Fig. S5 where the spatial profile of both pulses, for simplicity now treated as of rectangular shape, for different times is shown. As the time evolves the faster E_{IR} outpaces the slower E_{THz}. So, even if initially at a time $t = \tau$ no overlap was present, there will be a finite interaction time t_{I} between them. To estimate this quantity we assume the following shape for both pulses.

$$E_{\text{IR}} \propto \Theta(x + (ct/n_{\text{IR}} - \Delta x_{\text{IR}}/2)) - \Theta(x - (ct/n_{\text{IR}} + \Delta x_{\text{IR}}/2))$$

$$E_{\text{THz}} \propto \Theta(x + (ct/n_{\text{THz}} - \Delta x_{\text{THz}}/2)) - \Theta(x - (ct/n_{\text{THz}} + \Delta x_{\text{THz}}/2)) \tag{7}$$

Here Θ is the Heaviside function, c the speed of light and $\Delta x_{\text{IR}}, \Delta x_{\text{THz}}$ as well as $n_{\text{IR}}, n_{\text{THz}}$ the respective spatial widths and refractive indices of the pulses. When defining t_{I} as the time difference between i) the faster pulse reaching the lower one and ii) it fully outpacing the slower one, we can write $t_{\text{I}} = \dfrac{\Delta x_{\text{IR}} + \Delta x_{\text{THz}}}{c(1/n_{\text{IR}} - 1/n_{\text{THz}})}$. Δx_{IR} can be calculated by $\Delta x_{\text{IR}} = c\tau_{\text{laser}}/n_{\text{IR}}$ using the temporal pulse width of the laser τ_{laser}. In the same way the lower boundary for $\Delta x_{\text{THz}} = c\tau_{\text{laser}}/n_{\text{THz}}$ can be estimated. Together we arrive at $\tau_{\text{I}} = \tau_{\text{laser}} \dfrac{n_{\text{THz}} + n_{\text{IR}}}{n_{\text{THz}} - n_{\text{IR}}}$. Using the ordinary refractive indices presented in Fig. S4 results in $\tau_{\text{I}} \sim 2\,\tau_{\text{laser}}$. Together with the factor of $\sqrt{2}$ gained by the temporal autocorrelation of both pulses and a FWHM of the laser $\tau_{\text{laser}} = 300\,\text{fs}$ we get a minimal time resolution of $\sim 850\,\text{fs}$ which is in good agreement with the observed FWHM of ΔE in **??**b.

S7 Influence of detection bandwidth on fluence dependence

To investigate the influence of the detection scheme bandwidth on the fluence dependence, first $\Delta E(\tau)$ is obtained for $N = 1$ using EOS, as it has the highest available bandwidth ($\sim 1\,\text{THz}$, see Fig. S1). The obtained and normalized waveforms are displayed in Fig. S6. Note, that while in the plot only delays τ up to 25 ps are shown for clarity, each waveform was obtained up to $\tau = 120\,\text{ps}$ to also capture frequency components in the lower GHz regime.

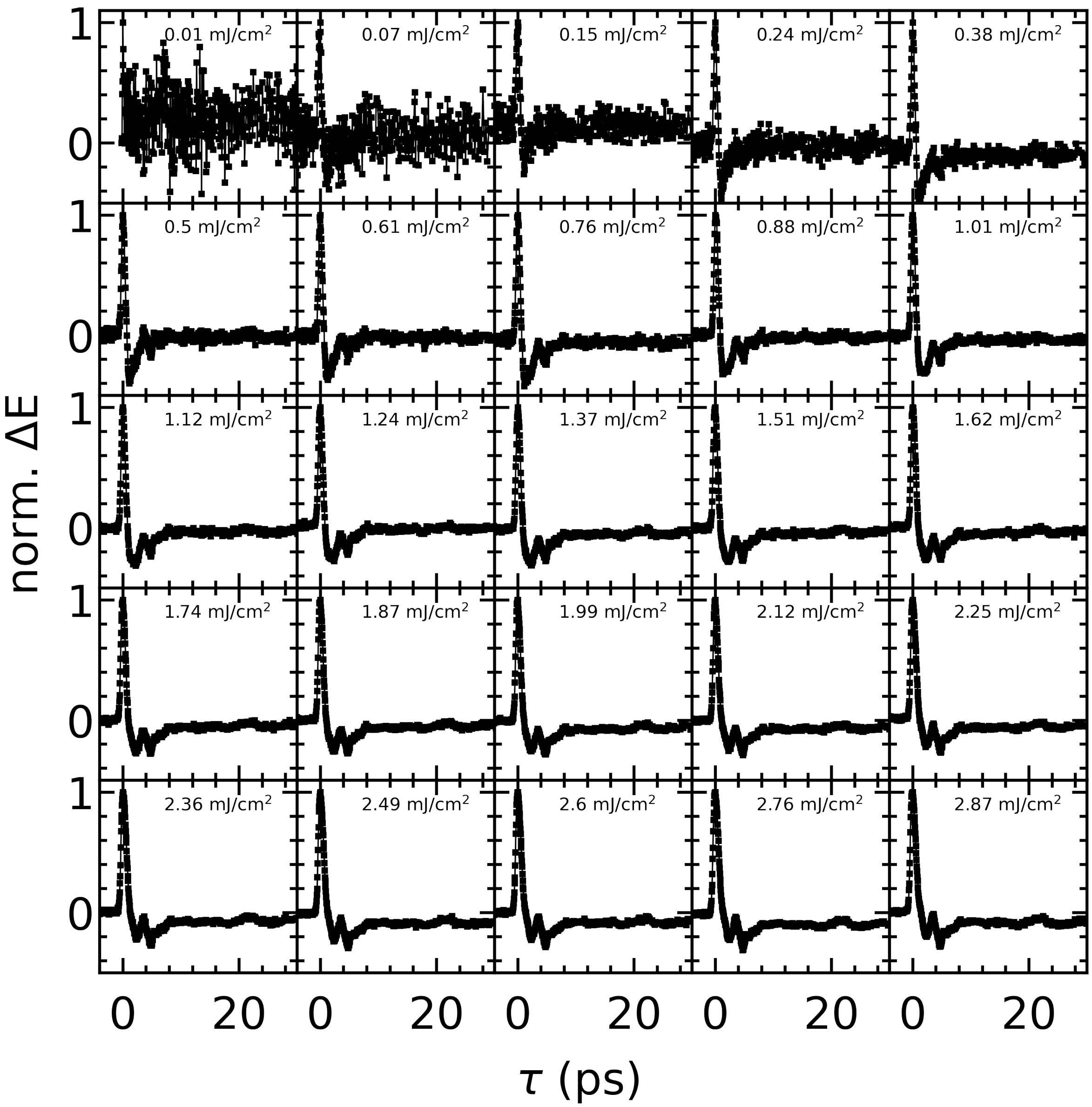

Figure S6: Waveforms ΔE for $N = 1$ obtained by EOS for different fluence Φ each normalized to its respective maximum. Note that the data extends up to delays of $\tau = 120\,\mathrm{ps}$ but for clarity each waveform is shown only up to 30 ps.

Next the FFT magnitude of each waveform in Fig. S6 is extracted and normalized using the following equation: norm. $\mathrm{FFT}(\Phi) = \mathrm{FFT}(\Phi)\ /\ \max(\mathrm{FFT}(\Phi_0))\ \cdot(\Phi_0/\Phi)$, with $\Phi_0 = 0.51\,\mathrm{mJ\,cm^{-2}}$. This procedures ensures that, if each frequency component scales linearly with Φ, each norm. $\mathrm{FFT}(\Phi)$ should look the same. As seen in Fig. S7 this is clearly not the case, as the frequency components $< 200\,\mathrm{GHz}$ grow faster for $\Phi > 0.77\,\mathrm{mJ\,cm^{-2}}$.

Furthermore, the higher Φ gets, the more dominant the low frequency components get.

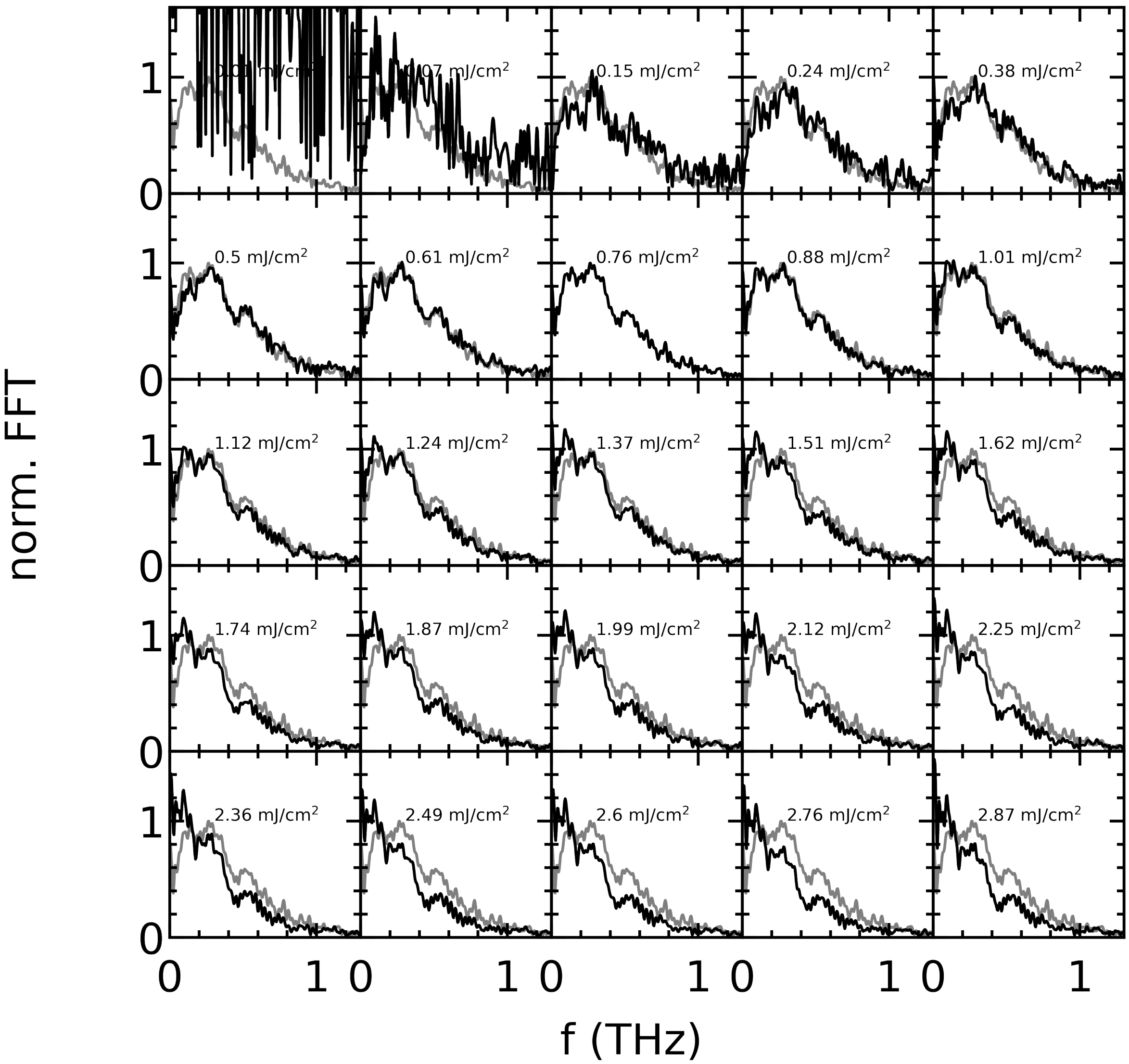

Figure S7: Magnitude of the FFT of each waveform shown in Fig. S6 normalized using the following equation: norm. $\mathrm{FFT}(\Phi) = \mathrm{FFT}(\Phi) \,/\, \max(\mathrm{FFT}(\Phi_0)) \cdot (\Phi_0/\Phi)$, with $\Phi_0 = 0.51\,\mathrm{mJ\,cm^{-2}}$. If each frequency component scales linearly with Φ, all panels should look the same. To easily see deviations form this behavior in each panel the norm. $\mathrm{FFT}(\Phi_0)$ is plotted as well (grey line). Note, that due to the normalization procedure the FFT in panel 1 for $\Phi \sim 0$ is highly exaggerated.

The detection scheme using the rf-tip in combination with the 50 GHz sampling oscilloscope is now mimicked by applying a fourth order, 50 GHz Butterworth filter, as seen in Fig. S1, to every FFT in Fig. S7 and using the same normalization as before. The resulting FFTs, depicted in Fig. S8, show this non-linear increase for frequencies below 50 GHz even

stronger.

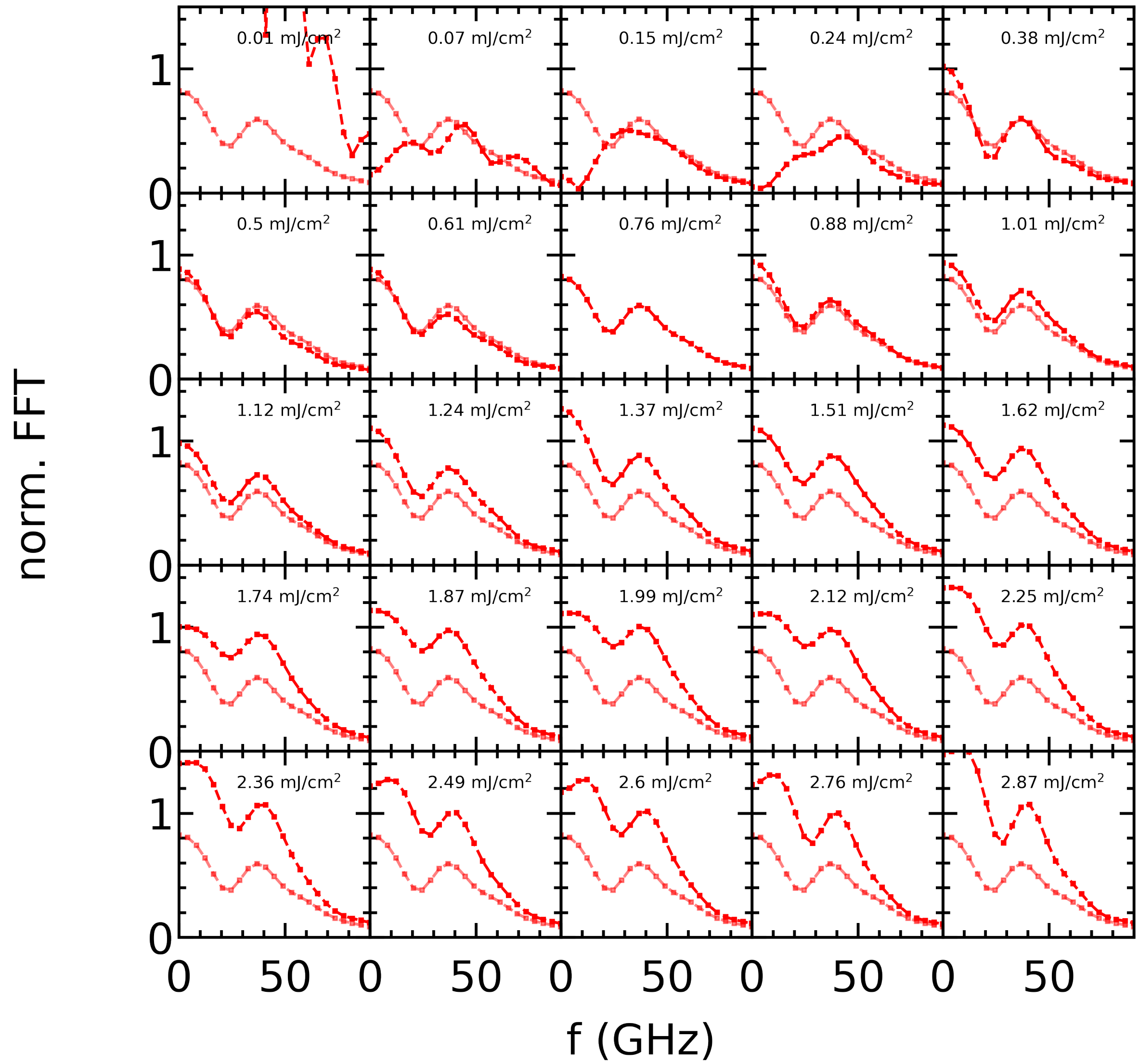

Figure S8: Magnitude of the FFT shown in Fig. S7 filtered with a Butterworth filter $G(f)$ of fourth and cutoff frequency of 50 GHz to see the fluence dependence of those frequency components, which are detectable with the tip. The same normalization procedure as in Fig. S7 is used after applying $G(f)$. For comparison the norm. FFT(Φ_0) is shown again in each panel (dashed light red).

To quantify the different behavior for THz and GHz bandwidth, respectively an averaged value of the FFT $\langle FFT \rangle$ for each Φ is calculated by integrating either the full spectrum (Fig. S7) or the filtered one (Fig. S8) over the whole frequency range. Together with the peak amplitude obtained either by EOS or the rf-tip they are plotted in Fig. S9, normalized

to their saturation value.

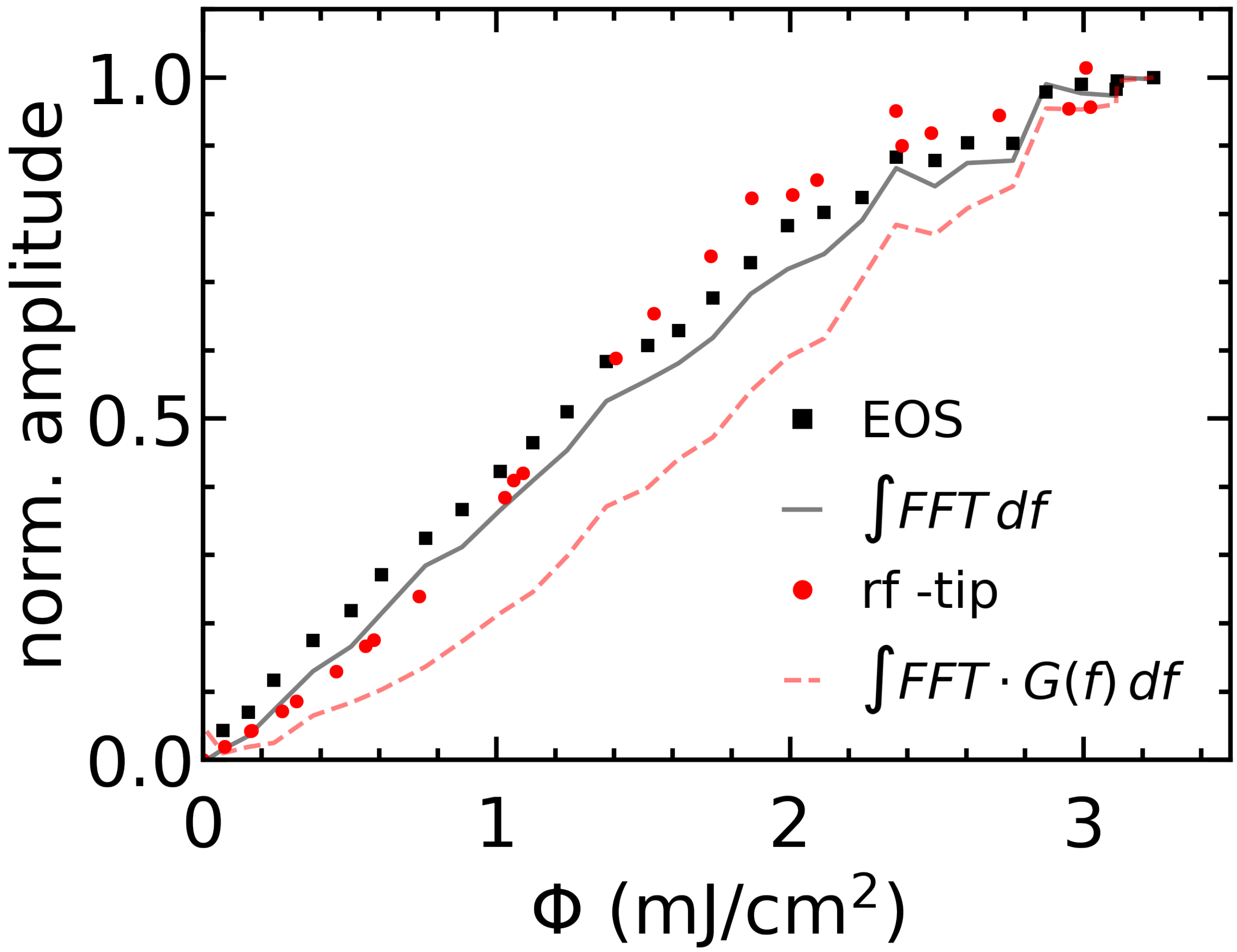

Figure S9: Amplitude over fluence, normalized to the saturation value, for $N = 1$ either using EOS (black squares) or the rf-tip (red circles). Φ for the rf-tip data was multiplied by 2.1 so the saturation plateaus for both techniques overlapp. Additionally, the averaged FFT $\langle FFT \rangle = \int FFT\,\mathrm{df}$ for either the full spectrum (black line, Fig. S7) or the filtered spectrum (dashed red line, Fig. S8) is plotted. For the rf-tip the substrate was Al_2O_3 and for EOS $LiNbO_3$.

When first comparing the values for peak amplitude and averaged FFT at THz bandwidth a good qualitative and quantitative agreement is visible, allowing the conclusion, that either quantity describes the fluence dependency in the same way; first, the signal increases linearly with increasing fluence and second, this increase slowly diminishes when reaching saturation fluence. Turning to the respective data at GHz bandwidth reveals only a qualitative alignment. For both, peak amplitude and averaged FFT, there is a non-linear increase of magnitude after exceeding a threshold fluence. Quantitatively however, said threshold fluence

and the steepness of the increase differ for both approaches. This may be explained by two things. First, the EOS can only resolve frequency components with a resolution $\sim 10\,\mathrm{GHz}$ for a total delay of $\sim 100\,\mathrm{ps}$ while the rf-tip is much more sensitive in this low frequency regime ($< 1\,\mathrm{GHz}$) as it can easily obtain waveforms for several ns. And these low frequency components dominate the signal obtained by the rf-tip and are missing in the analysis using EOS. Second, the choice of substrate for either measurement influences the behavior. For EOS sampling $LiNbO_3$ is required where as for the rf-tip Al_2O_3 was used, both having very different thermal conductivities. The thermal conductivity for $LiNbO_3$ ($\sim 4.5\,\mathrm{W\,m^{-1}\,K^{-1}}$) is nearly one order of magnitude smaller than for Al_2O_3 ($\sim 40\,\mathrm{W\,m^{-1}\,K^{-1}}$) thus the metal film is heated stronger when grown on $LiNbO_3$. As discussed in the main manuscript, the increased prominence of the low frequency components is suspected to be due the slower remagnetization for increased fluence, as the FM gets closer to its Curie temperature. So if the FM heats up faster, the non-linear behavior should start at a smaller fluence and increase then stronger. Indeed, this is the case in the presented data, (Fig. S9). In conclusion the different fluence behavior for EOS and rf-tip can be well explained in the framework of fluence dependent remagnetization and the resulting bandwidth dependence.

S8 Fluence dependence EOS waveguide

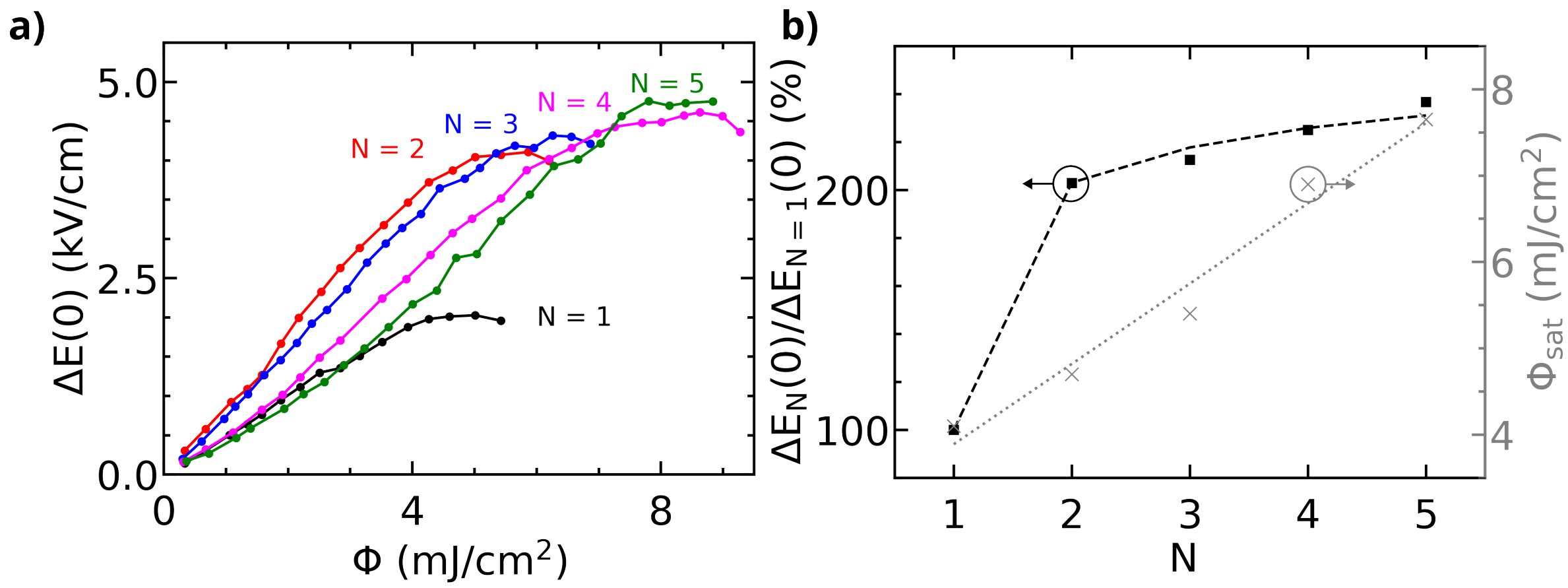

Figure S10: Amplitude $\Delta E(\tau = 0)$ for N = 1 to 5 with increasing fluence Φ obtained using EOS and optically probing the THz wave propagating inside the waveguide as seen in **??**. b) Amplitude $\Delta E_{\mathrm{N}}(0)$ (black) measured at saturation fluence Φ_{sat} (grey) for each N normalized to $\Delta E_{\mathrm{N=1}}(0)$. The black dashed line shows a fit of $\Delta E_{\mathrm{N}}(0)/\Delta E_{\mathrm{N=1}}(0)$ using **??** with $\alpha = 0.27$ and the impedance of our waveguide $R_{\mathrm{m}} = 40\,\Omega$. Additionally, a linear relation is fit to Φ_{sat} as guide for the eye (dotted grey line).

S9 Run time delay broadening

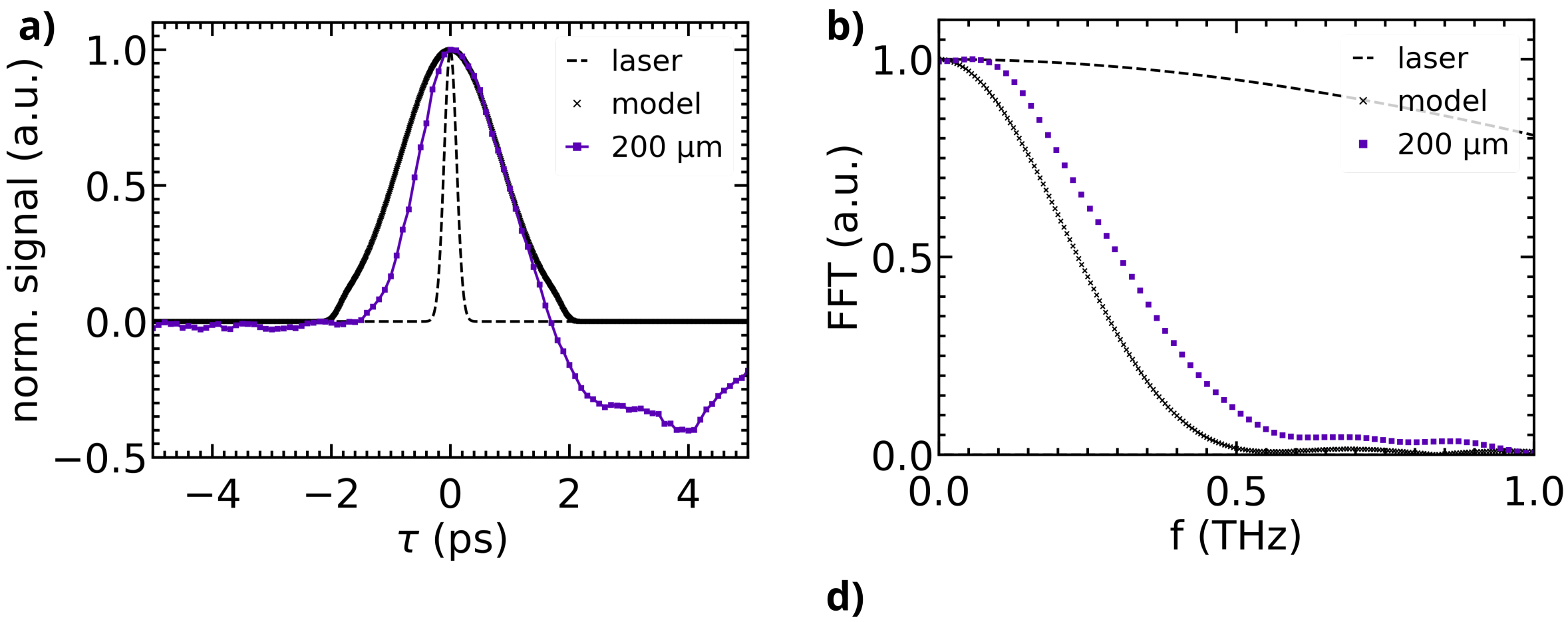

Figure S11: The influence of the finite emitter width and propagation velocity of the THz pulse inside the gold waveguide is investigated by using a simple runtime difference model (cross) and comparing it to data (squares) shown **??**. As reference the temporal shape/ spectrum of the fs laser pulse (dashed black line) is depicted in each graph. a) Time domain signal for an emitter width along y-direction of 250 µm, which corresponds to probing the pulse in the gap of the waveguide ($z = 200$ µm). b) shows the frequency spectrum of a). Each trace is normalized to its respective maximum.

To estimate temporal broadening of our pulses due to the run time delay across the emitter the following 1D model is utilized. When illuminating the emitter with the pump pulse, a charge current density $j_n = j_n^0(z)\, g(\tau_n)$ arises for all positions $|z_n| < w_{\text{emitter}}/2$. Here, w_{emitter} is the emitter width, $j_c^0(z_n)$ is the z-dependent amplitude of the charge current density, $g(\tau_n)$ the temporal shape of a single pulse and τ_n the time delay between appearance of j_n and its detection. All j_n sum up to the waveform j_{tot}:

$$j_{\text{tot}} = \sum_n j_n^0(z_n)\, g(\tau_n) \tag{8}$$

We assume that for the linear fluence regime $j_n^0(z)$ is given by the fluence profile of the pump pulse which has a $1/e^2$ radius of $r_{\text{pump}} = 110$ µm. As model function for $g(\tau_n)$ a unipolar gaussian with the $1/e^2$ pulse width of the laser pulse $\tau_{\text{pump}} = 250$ fs as the shortest possible pulse is chosen. For $z_0 = 0$ j_0 is shown in a) of Fig. S11 as dashed black line. With

the known average group velocity v_g we can write $\tau_n = \tau - z_n/v_g$ with τ being the absolute time delay. Together this forms:

$$j_n^0(z) = j_0 \exp\left(-2\frac{z^2}{r_\mathrm{pump}^2}\right) \qquad g(\tau_n) = \exp\left(-2\frac{(\tau - z_n/v_\mathrm{g})^2}{\tau_\mathrm{pump}^2}\right) \tag{9}$$

Inserting Eq. (9) in Eq. (8) and solving it numerically leads to the results in Fig. S11 a) with $w_\mathrm{emitter} = 250\,\mu\mathrm{m}$. As comparison the experimental determined waveform is shown there as well. Furthermore, in Fig. S11b the FFT of the waveforms is shown. In total the comparison is yielding an acceptable agreement. It should be noted, that only the positive values of the experimental data points were taken for the FFT to have a comparable unipolar waveform.

S10 Waveguide dispersion

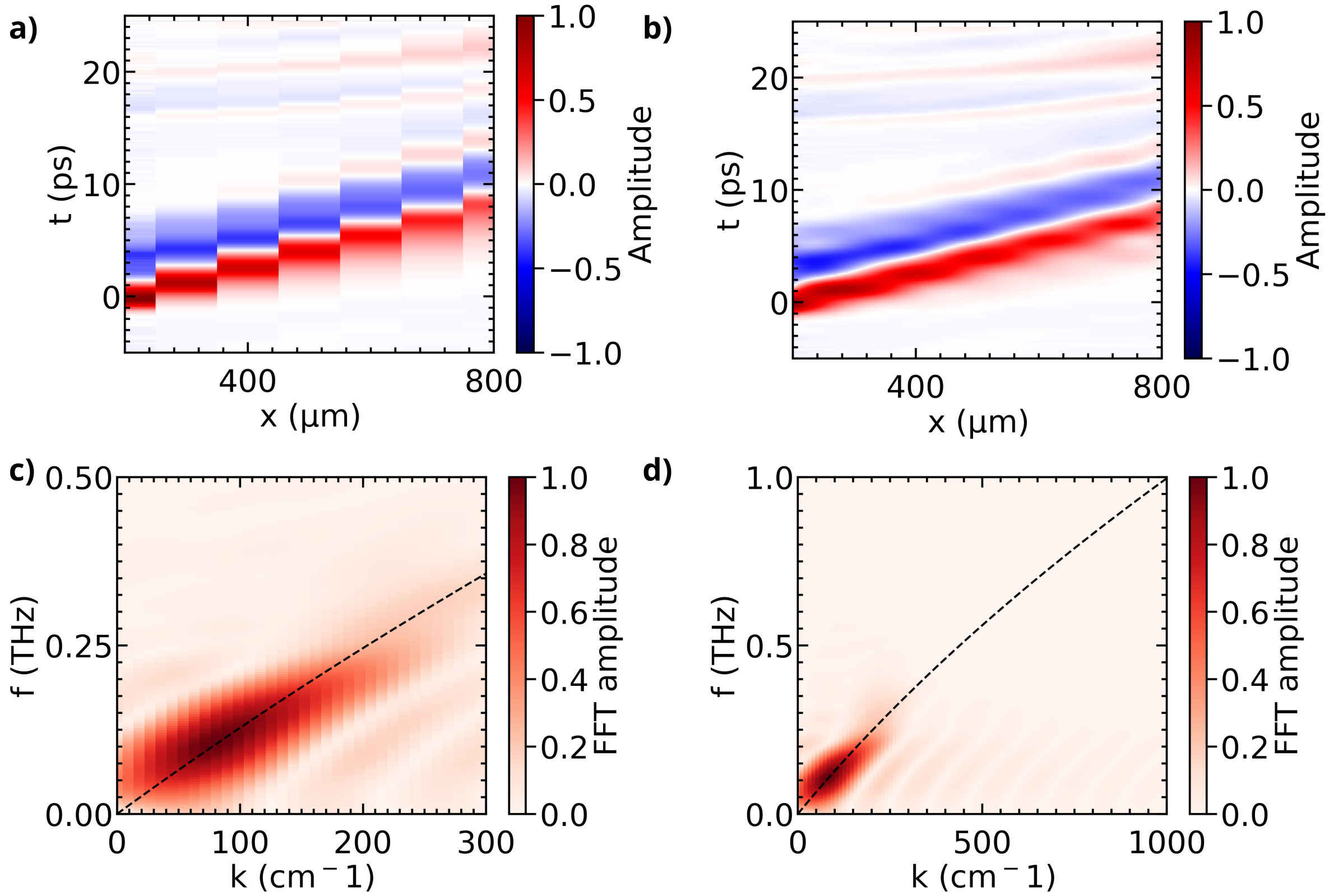

Figure S12: a) Raw and b) interpolated data from ?? represented as a normalized heat color map. c) and d) show the respective 2D-FFT of a) and b). The dashed lines correspond to the fitted dispersion relation $f(k)$ in Fig. S13 a).

A direct approach to obtain the dispersion relation $f(k)$ for a wavevector k in z-direction is to apply a two dimensional FFT to the spatially and temporally resolved data, as represented in Fig. S12. The dispersion obtained in this manner directly suffers from the large steps ($\sim 100\,\mu$m) made in our experiment.

Another way to obtain $f(k)$ is the following. Each waveform $\Delta E(z, \tau)$ can be written in the form of a Fourier series over all frequencies f as,

$$\Delta E(z, \tau) = \sum_f c_f \, \exp i\phi_f(z, \tau) \tag{10}$$

with complex amplitude c_{f} and total phase $\phi_f(z, \tau)$. Employing the FFT on $\Delta E(z, \tau)$ yields

for all f a real part a_f and imaginary part b_f. They are connected with c_f and ϕ_f in the following way:

$$c_\mathrm{f} = \sqrt{a_\mathrm{f}^2 + b_\mathrm{f}^2}, \qquad \tan(\phi_\mathrm{f}) = \frac{b_\mathrm{f}}{a_\mathrm{f}} \tag{11}$$

If ϕ_f is known for a set of z, e.g. by scanning $\Delta E(z,\tau)$ along the z-direction, for a given f the wavevector along z-direction k_f can be calculated by $k_\mathrm{f} = \partial_\mathrm{z}\phi_\mathrm{f}$. In our case for $z > 0$ it simplifies to an approximately linear behavior and thus to $k_\mathrm{f} = \frac{\Delta\phi_\mathrm{f}}{\Delta z}$. Doing so for all f yields the total dispersion $f(k)$ as depicted in Fig. S13a. The data was implicitly fit with $k = \kappa_0 f^2 + \kappa_1 f$ using $\kappa_0 = 2.5 \times 10^{-2}\,\mu\mathrm{m}^{-1}\,\mathrm{THz}^{-2}$ and $\kappa_2 = 7.5 \times 10^{-2}\,\mu\mathrm{m}^{-1}\,\mathrm{THz}^{-1}$.

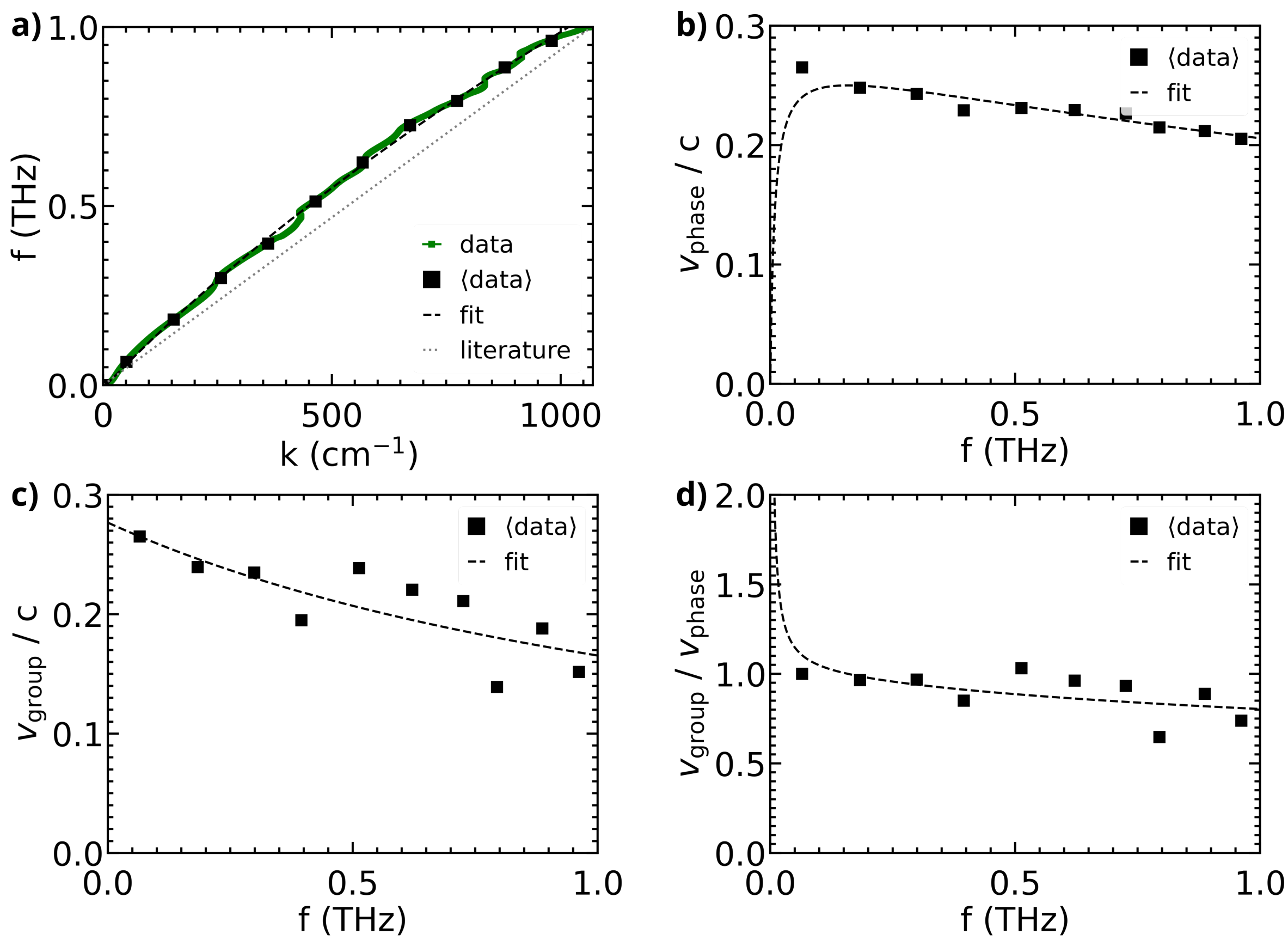

Figure S13: a) Dispersion f over k of our waveguide obtained using the waveforms displayed in ??. Implicitly, a square root behaviour $f \propto \sqrt{k}$ is fitted to the data. The raw data (green squares) is averaged (black squares), by binning the data using the smallest resolvable wavevector in the experiment $k = 2\pi/600\,\mu\mathrm{m} \sim 100\,\mathrm{cm}^{-1}$. As comparison, the dispersion found in literature ($2\pi f = c\,k/\sqrt{26}$[S2]) is plotted as grey dotted line. Using either the data or the fit from a) the frequency dependent b) phase velocity v_{phase} or c) group velocity v_{group} was calculated and normalized to the speed of light c. d) shows the ratio of v_{group} to v_{phase}.

With the help of $f(k)$ the f-dependent phase velocity v_{phase} and group velocity v_{group} can be calculated using $v_{\mathrm{phase}} = \frac{2\pi f}{k}$ and $v_{\mathrm{group}} = 2\pi\partial_k f$. This is either done numerically for the data points or analytically for the fit, and they are all together shown in Fig. S13b and Fig. S13c as well as the ratio of $v_{\mathrm{group}}/v_{\mathrm{phase}}$ in Fig. S13d.

References

(S1) Weis, R. S.; Gaylord, T. K. Lithium niobate: Summary of physical properties and crystal structure. *Applied Physics A Solids and Surfaces* **1985**, *37*, 191–203.

(S2) Kojima, S.; Nishizawa, S.; Kitahara, H.; Takeda, M. W. Complex Phonon-Polariton Dispersion of Congruent Lithium Niobate Studied by THz Time-Domain Spectroscopy. *Ferroelectrics* **2005**, *314*, 19–26.

(S3) Chelladurai, D.; Kohli, M.; Winiger, J.; Moor, D.; Messner, A.; Fedoryshyn, Y.; Eleraky, M.; Liu, Y.; Wang, H.; Leuthold, J. Barium titanate and lithium niobate permittivity and Pockels coefficients from megahertz to sub-terahertz frequencies. *Nature Materials* **2025**, *24*, 868–875.

(S4) Seifert, T.; Jaiswal, S.; Martens, U.; Hannegan, J.; Braun, L.; Maldonado, P.; Freimuth, F.; Kronenberg, A.; Henrizi, J.; Radu, I.; Beaurepaire, E.; Mokrousov, Y.; Oppeneer, P. M.; Jourdan, M.; Jakob, G.; Turchinovich, D.; Hayden, L. M.; Wolf, M.; Münzenberg, M.; Kläui, M.; Kampfrath, T. Efficient metallic spintronic emitters of ultrabroadband terahertz radiation. *Nature Photonics* **2016**, *10*, 483–488.

(S5) Cochard, C.; Spielmann, T.; Bahlawane, N.; Halpin, A.; Granzow, T. Broadband characterization of congruent lithium niobate from mHz to optical frequencies. *Journal of Physics D: Applied Physics 50*, 36LT01.
